# Viral-Mediated Microbe Mortality Modulated by Ocean Acidification and Eutrophication: Consequences for the Carbon Fluxes Through the Microbial Food Web

**DOI:** 10.3389/fmicb.2021.635821

**Published:** 2021-04-14

**Authors:** Andrea Malits, Julia A. Boras, Vanessa Balagué, Eva Calvo, Josep M. Gasol, Cèlia Marrasé, Carles Pelejero, Jarone Pinhassi, Maria Montserrat Sala, Dolors Vaqué

**Affiliations:** ^1^Biological Oceanography Laboratory, Austral Center for Scientific Research (CONICET), Ushuaia, Argentina; ^2^Department of Marine Biology and Oceanography, Institut de Ciències del Mar (CSIC), Barcelona, Spain; ^3^Center for Marine Ecosystems Research, School of Sciences, Edith Cowan University, Joondalup, WA, Australia; ^4^Institució Catalana de Recerca i Estudis Avançats (ICREA), Barcelona, Spain; ^5^Centre for Ecology and Evolution in Microbial Model Systems, Linnaeus University, Kalmar, Sweden

**Keywords:** ocean acidification, eutrophication, microbial food web, viral shunt, carbon fluxes

## Abstract

Anthropogenic carbon emissions are causing changes in seawater carbonate chemistry including a decline in the pH of the oceans. While its aftermath for calcifying microbes has been widely studied, the effect of ocean acidification (OA) on marine viruses and their microbial hosts is controversial, and even more in combination with another anthropogenic stressor, i.e., human-induced nutrient loads. In this study, two mesocosm acidification experiments with Mediterranean waters from different seasons revealed distinct effects of OA on viruses and viral-mediated prokaryotic mortality depending on the trophic state and the successional stage of the plankton community. In the winter bloom situation, low fluorescence viruses, the most abundant virus-like particle (VLP) subpopulation comprising mostly bacteriophages, were negatively affected by lowered pH with nutrient addition, while the bacterial host abundance was stimulated. High fluorescence viruses, containing cyanophages, were stimulated by OA regardless of the nutrient conditions, while cyanobacteria of the genus *Synechococcus* were negatively affected by OA. Moreover, the abundance of very high fluorescence viruses infecting small haptophytes tended to be lower under acidification while their putative hosts' abundance was enhanced, suggesting a direct and negative effect of OA on viral–host interactions. In the oligotrophic summer situation, we found a stimulating effect of OA on total viral abundance and the viral populations, suggesting a cascading effect of the elevated *p*CO_2_ stimulating autotrophic and heterotrophic production. In winter, viral lysis accounted for 30 ± 16% of the loss of bacterial standing stock per day (VMM_BSS_) under increased *p*CO_2_ compared to 53 ± 35% in the control treatments, without effects of nutrient additions while in summer, OA had no significant effects on VMM_BSS_ (35 ± 20% and 38 ± 5% per day in the OA and control treatments, respectively). We found that phage production and resulting organic carbon release rates significantly reduced under OA in the nutrient replete winter situation, but it was also observed that high nutrient loads lowered the negative effect of OA on viral lysis, suggesting an antagonistic interplay between these two major global ocean stressors in the Anthropocene. In summer, however, viral-mediated carbon release rates were lower and not affected by lowered pH. Eutrophication consistently stimulated viral production regardless of the season or initial conditions. Given the relevant role of viruses for marine carbon cycling and the biological carbon pump, these two anthropogenic stressors may modulate carbon fluxes through their effect on viruses at the base of the pelagic food web in a future global change scenario.

## Introduction

Current anthropogenic carbon release rates are unprecedented over the last 66 million years (Zeebe et al., 2016). Since preindustrial time until the present, the concentration of carbon dioxide (CO_2_) in the atmosphere has increased from ~277 ppm (e.g., Joos and Spahni, 2008) to 410 ppm in 2019 (Dlugokencky and Tans, 2020) due to the burning of fossil fuels, cement manufacturing, and land use changes. The ocean has absorbed about 30% of these CO_2_ emissions since 1750, leading to changes in seawater carbonate chemistry and a decline in pH (Sabine et al., 2004). Preindustrial pH values in the surface ocean of about 8.2 are expected to decrease to 7.8 at the end of the twenty-first century in a high-CO_2_-emission, “business-as-usual” scenario (Bopp et al., 2013), a process referred to as ocean acidification (OA, Caldeira and Wickett, 2003). During the past century, anthropogenic inputs of nitrogen (N) and phosphorus (P) to coastal ecosystems *via* river discharge have augmented mostly due to the use of fertilizers in agriculture (Galloway et al., 2004). Increased nutrient and organic matter supply to coastal environments has led to coastal eutrophication, which constitutes a major threat to ocean health (Rabalais et al., 2009). Thus, in addition to OA, eutrophication is another major global change pressure that directly affects marine ecosystems in the Anthropocene (Diaz and Rosenberg, 2008; Doney et al., 2009). Synergistic (Cai et al., 2011) or antagonistic interplays between both processes have been suggested (Borges and Gypens, 2010; Malone and Newton, 2020).

The microbial food web, composed of nano- and microalgae, autotrophic picoplankton, heterotrophic protists, heterotrophic bacteria and archaea, and virioplankton, constitutes the base of marine food webs and is a key component in the transfer of carbon and the regeneration of micro- and macronutrients (Azam, 1998). It has been estimated that ~50% of the global primary production is generated in the oceans by phytoplankton photosynthesis (Field et al., 1998). Heterotrophic prokaryotes contribute greatly to the degradation and transformation of organic matter through their extensive metabolic activity and act as an intermediate link between the dissolved organic carbon (DOC) pool and higher trophic levels through the microbial loop (Azam et al., 1983). On the other hand, marine viruses play a pivotal role in the carbon cycle of marine planktonic food webs (Suttle, 2005). Through killing the hosts and releasing the content of the cytoplasm to the environment, viruses transfer particulate organic matter (POM) to the dissolved organic matter (DOM) pool (Wilhelm and Suttle, 1999; Sheik et al., 2014). Mathematical models have shown that this so-called viral shunt has a negative effect on zooplankton production through its link to the microbial loop, especially under oligotrophic regimes (Murray and Eldridge, 1994). However, by fueling the DOM pool, viral activity also stimulates bacterial production (Malits and Weinbauer, 2009) with synergetic effects on the microbial food web (Berdjeb et al., 2011), boosts the recycling of nutrients (Gobler et al., 1997; Poorvin et al., 2004), and stimulates autotrophic production (Weinbauer et al., 2011) and bacterial respiration (Fuhrman, 1999; Middelboe and Lyck, 2002; Bonilla-Findji et al., 2008). Overall, viral lysis shifts the food web toward a more regenerative pathway, reducing the carbon flux to higher trophic levels (Brussaard et al., 2008b). Along with the lytic viral reproduction where the phage injects its genetic material into the host in order to redirect its metabolism toward the production of new phages and cell lysis, the genome of the phage may also remain in the host in a dormant stage and replicate along with the host (lysogenic cycle), until the lytic cycle is induced by stress or other unfavorable conditions (Weinbauer, 2004). The lytic cycle is also induced when bacteria grow actively (Wilson and Mann, 1997), and lysogeny has been shown to be important at high host densities (Knowles et al., 2016). Both life styles (lysis, lysogeny) are involved in maintaining genetic diversity among the host community (Weinbauer and Rassoulzadegan, 2004; Suttle, 2007; Malits and Weinbauer, 2009) having direct implications on the bulk community enzymatic machinery and, consequently, on global biogeochemical cycles.

Ocean microbes are currently exposed to large depth, regional, seasonal, and even daily pH variations driven by biological and physical processes, e.g., CO_2_ fixation during a phytoplankton bloom increases the pH in the water (Joint et al., 2011). For example, at the ocean time series station ALOHA, in the oligotrophic Central Pacific, the significant long-term decreasing trend of surface water pH by 0.04 pH units from 1988 to 2007 is superimposed to a seasonal surface pH variability of up to 0.06 pH units (Dore et al., 2009). In addition, microbes are exposed to pH values as low as those predicted in the surface oceans for the end of the twenty-first century in the thermocline, where sinking organic matter decomposition by aerobic respiration results in a reduction in pH (Joint et al., 2011). Moreover, in coastal systems, where increased nutrient loading and eutrophication stimulate microbial activity, the observed reductions in pH greatly exceed the values expected from anthropogenic CO_2_ uptake alone (Provoost et al., 2010). These observations suggest that, even if OA does not lead to dramatic changes in the biogeochemical cycles driven by microbes due to their flexibility to adjust to pH changes (Joint et al., 2011), coastal eutrophication can amplify the effects of pH change and thus affect biochemical processes.

Experimental studies have documented OA-induced changes in the phytoplankton community with shifts toward small picoplankton (Meakin and Wyman, 2011; Brussaard et al., 2013; Spilling et al., 2016; Crawfurd et al., 2017; Schulz et al., 2017), with major implications for the C fluxes in the marine planktonic food webs (Worden et al., 2015). However, mesocosm experiments in the Baltic Sea revealed that, in a picoplankton dominated food web, reduced plankton community respiration rates under high partial pressure of carbon dioxide (*p*CO_2_) did not translate into increased carbon export (Spilling et al., 2016).

Mesocosm experiments in the Arctic (Roy et al., 2013; Sperling et al., 2013; Zhang et al., 2013) and North Sea (Newbold et al., 2012; Oliver et al., 2014) did not find evidence of a *p*CO_2_ effect on the bulk prokaryotic community composition. However, the combined effects of acidification and nutrient additions (Baltar et al., 2015) or warming (Lindh et al., 2013) did select for specific bacterial phylotypes evidencing the synergistic effects of human-induced perturbations on marine systems. Bacterial bulk production, enzyme activity, and growth rate were indeed enhanced under future atmospheric *p*CO_2_ levels (Grossart et al., 2006; Sala et al., 2016).

The effect of changes in *p*CO_2_/pH on lytic viral production and viral abundance has been less studied, and the few reports available so far provide contradictory results. Classically studied *Escherichia coli* bacteriophages such as T2 and T7 are indeed sensitive to changes in pH (Danovaro et al., 2011). Elevated *p*CO_2_ did not alter total viral abundance in mesocosm experiments in the North Sea (Rochelle-Newall et al., 2004) and the Arctic Ocean (Brussaard et al., 2013) but increased viral abundances in a Baltic Sea mesocosm study (Tsiola et al., 2017). In a mesocosm experiment, large (i.e., high fluorescence) virus abundance was higher in the control compared to elevated *p*CO_2_, and two specific large double-stranded DNA (dsDNA) viruses infecting the haptophytes *Emiliania huxleyi* and *Crysochromulina ericina* decreased in abundance with increasing *p*CO_2_ levels, suggesting changes in viral diversity (Larsen et al., 2008). Other studies in virus–host systems have shown a negative effect of OA on primary production of *Synecchococcus* and on the infection by its associated virus (Traving et al., 2013) but no effects on viral lysis of *Micromonas pulsilla* (Maat et al., 2014) with a potential positive feedback for carbon and nutrient cycling. No discernable effects of enhanced *p*CO_2_ on lytic viral production could be detected in other acidification mesocosm experiments in the Baltic Sea (Crawfurd et al., 2017), Mediterranean (Tsiola et al., 2017), and Arctic Ocean (Vaqué et al., 2019), while lysogeny increased with OA in combination with warming in that Arctic Ocean study (Vaqué et al., 2019). Nonetheless, the effect of decreased pH on lysogeny remains uncertain.

Thus, no conclusive data on the effect of ocean acidification on virioplankton and the consequences for microbial food web carbon fluxes exist, even less in combination with eutrophication. In this context, we conducted two mesocosm acidification experiments with microbial communities of the Mediterranean, in which we also added excess nutrients, in two different seasons, winter and summer, to investigate the combined effect of OA and eutrophication on the microbial food web. These experiments already demonstrated a shift to medium-sized phytoplankton under acidified but oligotrophic conditions (Sala et al., 2016), eutrophication effects on dissolved organic matter quality and composition regardless of changes in pH (Aparicio et al., 2016), synergistic effects of nutrient loading and OA on prokaryotic community structure (Baltar et al., 2015), and OA effects on prokaryoplankton gene expression (Bunse et al., 2016). In the present study, we followed viral abundances along with those of their hosts and assessed phage-mediated bacterial mortality and lysogeny in these mesocosm experiments in order to evaluate the potential implications of anthropogenic perturbations of marine systems on viruses and viral-mediated processes in the microbial food web.

## Materials and Methods

Two experiments (referred to as WINTER and SUMMER hereafter) were performed with water collected from the Blanes Bay Microbial Observatory (BBMO), NW Mediterranean (Gasol et al., 2016) on February 17, 2010 and on July 6, 2011, respectively. For both experiments, surface water samples were mixed and quickly transferred to eight 200-L polyethylene mesocosms at the Institut de Ciències del Mar aquaria facilities in Barcelona (corresponding to day 0 of the experiments). Four experimental conditions were randomly assigned to duplicated containers: KB (control), KA (lowered pH), NB (nutrient amended), and NA (nutrient amended and lowered pH). Experiments were conducted in a temperature-controlled chamber, set at approximately *in situ* temperature (Table 1), and a combination of cool-white and grolux lamps was used to illuminate the mesocosms. Measured light intensity inside the containers was 121.3 ± 3.5 μmol m^−2^ s^−1^ in the WINTER and 140.8 ± 13.5 μmol m^−2^ s^−1^ in the SUMMER experiments. The light/dark cycle was set at 12:12 h. Further details can be found in Sala et al. (2016), Baltar et al. (2015), Aparicio et al. (2016), and Bunse et al. (2016).

**Table 1:** *In situ* and experimental conditions in the mesocosms after adding nutrients in experiments WINTER and SUMMER.

**WINTER**	**Temp. (°C)**	**Light (μmol m^−2^ s^−1^)**	**${\text{NO}}_{3}^{-}$ + ${\text{NO}}_{2}^{-}$ (μM)**	**${\text{PO}}_{4}^{\text{3}-}$ (μM)**	**${\text{SiO}}_{4}^{\text{4}-}$ (μM)**	**pH**
*In situ*	13	110	2.43	0.11	2.49	8.05 ± 0.00
KB	14 ± 1	121.3 ± 3.5	2.87 ± 0.12	0.11 ± 0.01	1.73 ± 0.07	7.98 ± 0.02
KA	14 ± 1	121.3 ± 3.5	2.79	0.17 ± 0.02	2.29 ± 0.13	7.80 ± 0.02
NB	14 ± 1	121.3 ± 3.5	17.43	1.19 ± 0.04	31.40 ± 0.32	7.99 ± 0.01
NA	14 ± 1	121.3 ± 3.5	17.35 ± 0.63	1.10 ± 0.06	31.42 ± 1.33	7.82 ± 0.01
**SUMMER**						
*In situ*	22	1,001	0.05	0.03	0.52	8.07 ± 0.00
KB	22 ± 1	249.5 ± 0.7	0.94 ± 0.07	0.04 ± 0.00	0.51 ± 0.02	8.01 ± 0.00
KA	22 ± 1	232.5 ± 3.5	0.37 ± 0.06	0.03 ± 0.00	0.57 ± 0.04	7.76 ± 0.05
NB	22 ± 1	242.0 ± 1.4	4.91 ± 0.6	0.26 ± 0.02	7.22 ± 0.15	8.03 ± 0.02
NA	22 ± 1	239.0 ± 31.1	4.94 ± 0.3	0.27 ± 0.01	7.36 ± 0.01	7.76 ± 0.02

*Nutrient data are the average of two replicates ± range. For ${\text{NO}}_{3}^{-}$+${\text{NO}}_{2}^{-}$ and the KA and NB conditions in WINTER, only one of the replicates was measured*.

Nitrogen and phosphorus were added at Redfield ratio, while silicate was added in excess to ensure diatom growth (the final molar ratio for P/N/Si was ~1:16:30) before the lights were turned on, on February 18 and July 7, for experiments WINTER and SUMMER, respectively (corresponding to day 1). In KA and NA, pH was artificially lowered by bubbling, in a controlled way, small amounts of CO_2_ (99.9% purity) directly into the mesocosms. This was done every morning in order to maintain the levels of pH in the acidified tanks at around 0.25–0.30 pH units lower than the controls (KB and NB), which were bubbled with equivalent amounts of pure air to ensure similar turbulent conditions. The lowering in pH of the acidified treatments vs. the controls is equivalent to the values projected for the end of the twenty-first century following relatively pessimistic scenarios (Bopp et al., 2013). Seawater pH in the mesocosms was continuously monitored using glass electrodes (LL Ecotrode plus—Metrohm, calibrated every day with a Tris buffer, following standard procedures, Dickson et al., 2007) and recorded by a D130 data logger (Consort, Belgium). In addition, prior to each controlled addition of CO_2_, we performed precise measurements of the mesocosm's seawater pH using spectrophotometry (Clayton and Byrne, 1993) and alkalinity through a fast, single-point potentiometric titration (Pérez et al., 2000). Initial conditions are summarized in Table 1. To avoid sedimentation, we gently agitated the water during 5 min twice a day. Samples for chlorophyll, microbial abundances, and prokaryotic activity were taken daily (for details see Sala et al., 2016), while experiments for virus-mediated mortality of heterotrophic prokaryotes and the fraction of lytic and temperate viruses were performed at days 1, 5, and 8 for the WINTER and at days 0, 4, and 8 for the SUMMER experiment.

### Inorganic Nutrients and Chlorophyll *a*

Samples for inorganic nutrients were kept frozen at −20°C until analysis, which were performed using a CFA Bran+Luebbe autoanalyzer following the methods described by Hansen and Koroleff (2007).

For chlorophyll *a* (chl *a*) analysis according to Yentsch and Menzel (1963), 50 ml of seawater was filtered through Whatman GF/F filters. Pigments were extracted in 90% acetone at 4°C for 24 h and determined by measuring their fluorescence using a Turner Designs fluorometer.

### Microbial Abundances

For picophytoplankton, fresh, unstained samples were analyzed in a Becton Dickinson FACSCalibur flow cytometer at high speed (about 100 μl min^−1^) following Marie et al. (2001). Phototrophic populations (*Prochlorococcus, Synechococcus*, small and large picoeukaryotes) were discriminated and enumerated according to their light scatter and specific autofluorescence properties.

Since the experiment took place with surface waters that do not harbor almost any Archaea (Alonso-Sáez et al., 2007), we are using the term bacteria to indicate both Bacteria and Archaea. Samples for heterotrophic bacterial abundance (BA) were fixed with paraformaldehyde (1%) and glutaraldehyde (0.05%), kept at room temperature for about 10 min and then flash frozen in liquid nitrogen. Within a few days, the samples were thawed, stained with SYBR Green I (Molecular Probes Inc.) for 10 min, and analyzed in a Becton Dickinson FACSCalibur flow cytometer as described previously (Marie et al., 1997; Gasol and Morán, 2015). Bacteria were determined in plots of 90° light scatter (SSC) vs. green DNA fluorescence (FL1). Differences in FL1 allowed to separate bacteria with low nucleic acid content (LNA) from those with high nucleic acid content (HNA) (Gasol et al., 1999).

### Viral Abundances

For the abundance of virus-like particles (VLPs), samples (1 ml) were fixed with 0.2-μm-filtered glutaraldehyde (0.5% final concentration), incubated at 4°C for 15–30 min, and subsequently frozen in liquid nitrogen and stored at −80°C. Upon thawing, viruses were stained with SYBR Green I (Molecular Probes Inc.) for 10 min in the dark at 80°C and quantified after dilution with TE buffer [10 mM Tris, 1 mM ethylenediaminetetraacetic acid (EDTA), pH = 8] using flow cytometry and an optimized protocol (Marie et al., 1999; Brussaard, 2004). Four VLP populations were distinguished based on their signature in the cytometric plots of side scatter (SSC) vs. green fluorescence (FL1): low, medium, high, and very high green fluorescence VLPs (Supplementary Figure 1). The latter group was identified by its cytometric signature comparable to viruses infecting *Pyramimonas orientalis* and *Phaeocystis pouchetii* (Brussaard, 2004). The low and medium green fluorescence subpopulations represent mostly the numerically dominant bacteriophages (Brussaard, 2009). The sample flowrate was accurately calibrated following the protocol of Marie et al. (2001) and used to calculate the abundances of viruses.

### Bacterial Activity

Bulk heterotrophic bacterial production (BP) was estimated from ^3^H-leucine incorporation (Kirchman et al., 1985). For each sample, quadruplicate aliquots (1.2 ml) and two trichloroacetic acid (TCA)-killed controls were incubated with 40 nM ^3^H-leucine for about 1.5 h in the dark at *in situ* temperature. Subsequently, leucine incorporation was stopped by adding 120 μl of cold TC 50% to each replicate. Samples were stored at −20°C until processed following published methods (Smith and Azam, 1992). Leucine incorporated into bacterial biomass was converted to bacterial carbon production using the theoretical factor of 1.55 kg C mol^−1^ Leu that assumes no isotope dilution (Simon and Azam, 1989).

The numbers of actively respiring bacteria were determined using the fluorogenic tetrazolium dye 5-cyano-2,3-ditolyl tetrazolium chloride (CTC) labeling of highly active cells (Sherr et al., 1999; Sieracki et al., 1999). CTC was added to 1-ml subsamples at a final 5 mM concentration from a daily prepared 10× batch and incubated at *in situ* temperature for 3 h. After incubation, the samples were analyzed with a FACSCalibur flow cytometer as described in detail elsewhere (Gasol and Arístegui, 2007).

### Lytic Viral Production, Fraction of Infected Cells and Lysogens, Viral-Mediated Mortality, and C Release Rates

Lytic viral production (VP_L_), the fraction of infected cells (FIC), induced viral production from lysogens (VP_I_), and the fraction of lysogenic cells (FLC) were estimated for selected samples using the virus reduction approach (VRA, Weinbauer et al., 2010). The underlying principle of the VRA is to reduce viral abundances in the water, thereby essentially preventing new viral infections. Thus, it is assumed that the viruses produced during the incubation originate from already infected cells. In order to eliminate viruses, bacteria in ~400 ml from one replicate of each treatment were concentrated using a tangential flow system with a peristaltic pump (Watson-Marlow 323) equipped with a 0.2-μm cartridge (VIVAFLOW 200). To obtain virus-free seawater, the 0.2-μm pore-size ultrafiltrate was passed through a 30-kDa cartridge (VIVAFLOW 200). The bacterial concentrates were brought up to the original volume with virus-free seawater and incubated in triplicates in 50-ml Falcon tubes (BD Biosciences) at *in situ* temperature and in the dark (Table 1) for 24 h. At time 0 of these VRA experiments, three additional tubes were amended with mitomycin C (MC, Sigma) at a final concentration of 1 μg ml^−1^ in order to induce the lytic cycle of lysogens; untreated samples served as controls (Paul and Weinbauer, 2010). Subsamples (1 ml) for VA and BA from each incubation were taken every 2–4 h during the first 6–12 h and after 24 h, fixed with glutaraldehyde (0.5% final concentration), incubated at 4°C for 15–30 min, subsequently frozen in liquid nitrogen, and stored at −80°C until counted by flow cytometry as described above. VP_L_ was calculated as the increase in viral abundance over short time intervals (~4 h). An increase in viral abundance in the MC treatments represents VP_L_ + VP_I_ (Paul and Weinbauer, 2010; Weinbauer et al., 2010). VP_L_ and VP_I_ were corrected for the changes in the initial BA in the viral production assays with respect to *in situ* BA. The burst size (BS) was estimated following the approach of Wells and Deming (2006), i.e., dividing the number of viruses produced during the first hour of incubation by the concomitant decline of bacterial abundance. Dividing the number of produced phages by BS yields the number of lysed cells and gives an estimate of the fraction of infected cells (FIC) when divided by BA at the start of the experiments. Dividing the number of induced phages by BS and the BA at t0 gives an estimate of FLC. To obtain the rate of cell lysis, viral production corrected for *in situ* bacterial abundance was divided by the average estimated BS for WINTER and SUMMER, respectively. Lysis rates were used to calculate virus-mediated mortality of bacteria per day as a percentage of the bacterial standing stock (VMM_BSS_ day^−1^). Carbon release rates through viral lysis were calculated based on VP by converting the number of lysed bacteria into carbon using a factor of 20 fg C cell^−1^ (Lee and Fuhrman, 1987).

### Statistics

All statistical analyses were performed with JMP 7.0 (SAS). The Shapiro–Wilk *W*-test was used to check for normal distribution of data. Analysis of covariance (ANCOVA) with time as a covariate was used to discern the effects of time from those of the lowered pH and nutrient amendment for time-averaged parameters. Kruskal–Wallis tests for non-normal distributions were used to evaluate the acidification effect for K and N treatments, separately. Spearman rank correlation for non-parametric data was performed to determine the relationships between the various measured parameters.

## Results

### Experimental Conditions

**Figure 1:**
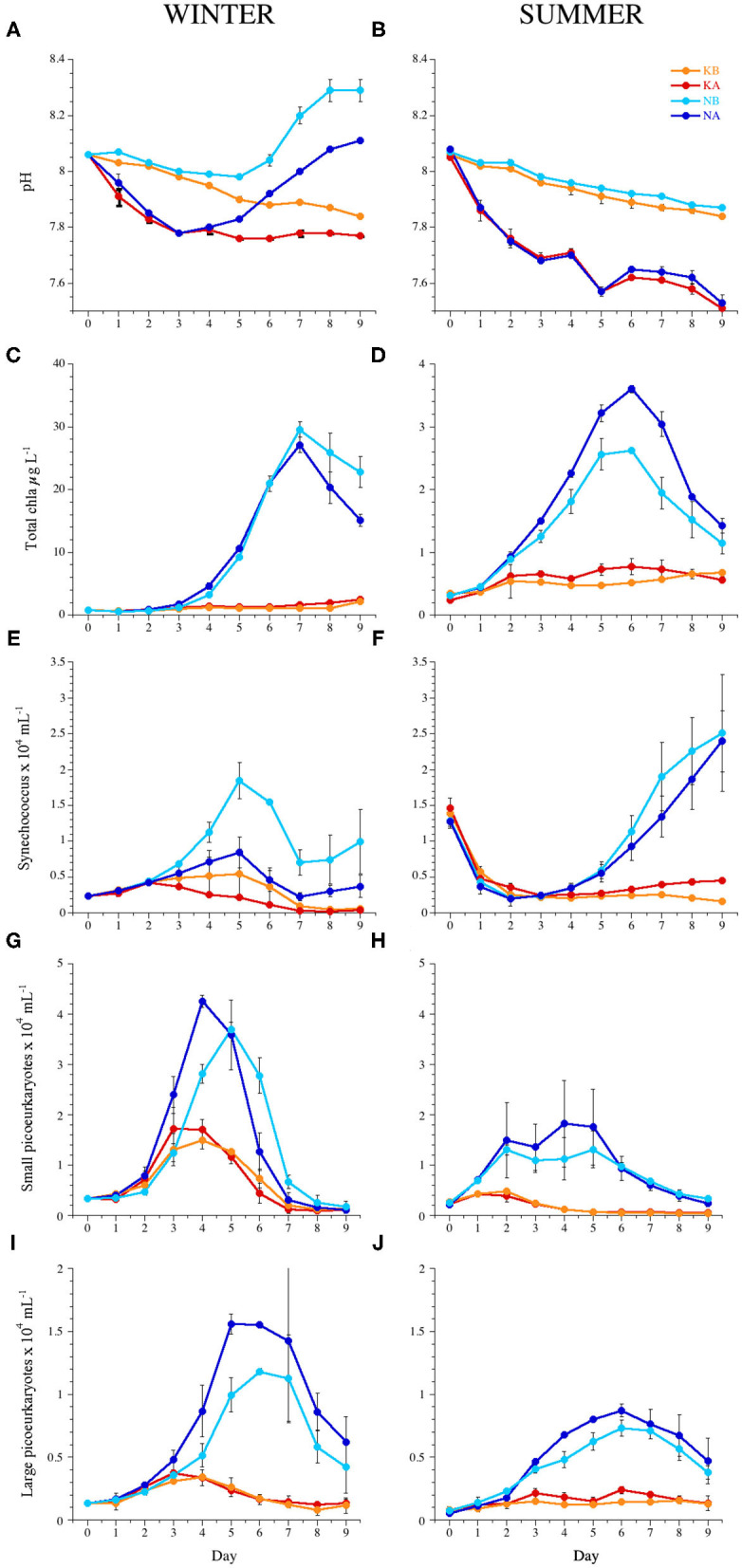
Temporal dynamics of **(A,B)** pH, **(C,D)** total chlorophyll *a*, **(E,F)** abundance of *Synechococcus* sp., **(G,H)** small picoeukaryotes, and **(I,J)** large picoeukaryotes during the experiments WINTER (left) and SUMMER (right). Bars indicate the maximum and minimum values of experimental duplicates; when not visible, they are contained within the symbol. Note the differences in the y-axis scales of chlorophyll *a* between seasons. KB, control; KA, lowered pH; NB, nutrient amended; NA, nutrient amended and lowered pH.

Initial temperature, light conditions, pH, and the concentrations of inorganic nutrients for the WINTER and SUMMER experiments are summarized in Table 1. For a detailed description of the experimental conditions and microplankton and bacterial dynamics in the experiments, see Baltar et al. (2015), Sala et al. (2016), and Aparicio et al. (2016). In WINTER, *in situ* nutrient concentrations (phosphate and silicate) were four to five times higher than in SUMMER. Initial experimental nitrogen concentrations in the N treatments were higher in the WINTER than in the SUMMER experiment, as they were added by multiplying the monthly average concentration measured in the BBMO during the last 10 years by a factor of 8 (Table 1). Due to the biological activity in the mesocosms, the pH in the experimental chambers of the WINTER experiment tended to increase with time and varied by up to 0.12 U (7.8 ± 0.05 in the KA, 7.9 ± 0.07 in the KB, 7.9 ± 0.12 in the NA, and 8.1 ± 0.12 in the NB treatments, Figure 1A). On the contrary, during SUMMER, the pH tended to decrease and was less variable (7.6 ± 0.08 in the KA, 7.9 ± 0.06 in the KB, 7.6 ± 0.07 in the NA, 7.9 ± 0.05 in the NB treatments, Figure 1B).

### Chlorophyll a Concentration and Autotrophic Picoplankton

Average chl *a* concentration was six times higher in the WINTER (6.8 ± 8.9 μg L^−1^) than in the SUMMER experiment (1.1 ± 0.9 μg L^−1^, *n* = 159, *P* < 0.0001) and increased significantly in the nutrient-amended treatments with time and with respect to the controls (Table 2). Chl *a* peaked on days 7 and 6 in the WINTER and the SUMMER experiment, respectively, being almost 10 times higher in WINTER than in SUMMER (Figures 1C,D). In WINTER, chl *a* concentration increased significantly with acidification only in the K treatments (Table 3), while in SUMMER, chl *a* concentration was significantly stimulated in the low pH treatments, regardless the nutrient conditions (Figure 1D and Table 2).

**Table 2:** Results of an analysis of covariance (ANCOVA) with time as a covariate showing the significance of the effects of acidification and nutrient amendments during the time course (days 2–9) of the experiments WINTER and SUMMER.

	**WINTER**						**SUMMER**					
**Variable**	***n***	**Time**	**Lower pH**		**Nutrients**		***n***	**Time**	**Lower pH**		**Nutrients**	
VLP	64	<0.0001		ns	+	<0.0001	64	<0.0001		ns	+	<0.0001
Low VLP	64	<0.0001		ns	+	<0.0001	64	<0.0001		ns	+	<0.0001
Medium VLP	64	ns		ns	+	0.0070	64	<0.0001		ns	+	0.0085
High VLP	64	ns	+	0.0022	+	<0.0001	64	<0.0001		ns	+	0.0215
Very High VLP	58	<0.0001		ns		ns						
BA	64	<0.0001		ns		ns	64	<0.0001		ns	+	<0.0001
BP	64	<0.0001		ns	+	0.0027	64	ns		ns	+	<0.0001
% CTC+ cells	40	<0.0001		ns		ns	58	ns		ns	+	<0.0001
Chl *a*	64	<0.0001		ns	+	<0.0001	63	ns	+	0.0316	+	<0.0001
*Synechococcus*	64	0.0492	−	0.0002	+	<0.0001	63	<0.0001		ns	+	<0.0001
*Prochlorococcus*	64	<0.0001		ns	+	0.0134	62	0.0094		ns	+	0.0001
Small picoeuka	64	<0.0001		ns	+	0.0019	63	<0.0001		ns	+	<0.0001
Large picoeuka	64	ns		ns	+	<0.0001	63	ns	+	0.0217	+	<0.0001

*ns, not significant; VLP, total abundance of virus-like particles; low, medium, high, very high VLP, VLP subpopulations with low, medium, high, or very high green fluorescence in the cytometric signature, respectively (Supplementary Figure 1); BA, bacterial abundance; BP, bacterial heterotrophic production; % CTC+ cells, the percentage of CTC-labeled cells; chl a, chlorophyll a; picoeuka, autotrophic picoeukaryotes*.

**Table 3:** Spearman's rank correlation coefficients (ρ) and corresponding significance (*P*) of measured pH in the experimental mesocosm with other parameters shown for the experiments WINTER (*n* = 12 for VP_L_ and VMM, *n* = 24 for CTC+ and *n* = 36 for the other parameters) and SUMMER (*n* = 14–15 for VP_L_ and VMM, *n* = 26 for LNA and HNA, and *n* = 36 for the other parameters).

	**WINTER K**		**WINTER N**		**SUMMER K**		**SUMMER N**	
**Parameters**	**ρ**	***P***	**ρ**	***P***	**ρ**	***P***	**ρ**	***P***
VLP	−0.258	*ns*	**0.390**	0.0187	**−0.555**	0.0004	**−0.539**	0.0007
Low VLP	–**0.371**	0.0258	**0.520**	0.0012	**−0.528**	0.0009	**−0.569**	0.0003
Medium VLP	0.124	*ns*	0.073	*ns*	**−0.414**	0.0122	**−0.519**	0.0012
High VLP	−0.050	*ns*	0.183	*ns*	**−0.562**	0.0004	**−0.579**	0.0002
Very High VLP	**−0.422**	0.0145	**0.698**	<0.0001	–	–	–	**–**
VP_L_	**0.713**	0.0090	0.089	*ns*	−0.022	*ns*	0.577	*ns*
VMM _BSS_	0.389	*ns*	**0.633**	0.0370	0.389	*ns*	0.866	*0.057*
C release d^−1^	**0.713**	0.0090	0.089	*ns*	−0.022	*ns*	0.577	*ns*
BA	0.049	*ns*	**−0.426**	0.0096	**−0.481**	0.0030	**−0.399**	0.0159
LNA	−0.313	*ns*	0.301	ns	**0.394**	0.0466	0.197	*ns*
HNA	0.068	*ns*	**−0.464**	0.0044	−0.034	*ns*	**−0.392**	0.0479
BP	0.152	*ns*	−0.221	*ns*	0.087	*ns*	0.173	*ns*
% CTC+ cells	0.012	*ns*	**−0.523**	0.0087	0.240	*ns*	0.171	*ns*
chl *a*	**−0.681**	<0.0001	**0.453**	0.0055	**−0.656**	<0.0001	**−0.428**	0.0091
*Synechococcus*	**0.504**	0.0017	−0.087	*ns*	**−0.341**	0.0421	**−0.394**	0.0193
*Prochlorococcus*	0.276	*ns*	**−0.669**	<0.0001	0.002	*ns*	**−0.429**	0.0101
Small picoeuka	0.202	*ns*	**−0.714**	<0.0001	**0.373**	0.0251	0.239	*ns*
Large picoeuka	0.110	*ns*	−0.116	*ns*	**−0.478**	0.0032	**−0.459**	0.0055

*Notice that a positive ρ means a negative effect of acidification*.

*ns, not significant; VLP, total abundance of virus-like particles; low, medium, high, very high VLP, VLP subpopulations with low, medium, high, or very high green fluorescence in the cytometric signature, respectively; VP_L_, lytic viral production; VMM_SS_ day^−1^, viral-mediated loss of bacterial standing stock per day: BA, bacterial abundance; LNA and HNA, bacteria with low and high fluorescence in the cytometric signature, respectively; BP, bacterial heterotrophic production; % CTC+ cells, the percentage of CTC-labeled cells; chl a, chlorophyll a; picoeuka, autotrophic picoeukaryotes. Bold numbers denote significant effects of acidification; positive effects when highlighted green and negative effects when highlighted red*.

Autotrophic picoplankton growth was stimulated by nutrient addition anticipating the chlorophyll peak at least in WINTER (Figure 1 and Table 2). Cynanobacteria of the genus *Synechococcus* were negatively affected by acidification in WINTER and not affected in SUMMER (Figures 1E,F and Table 2). *Prochlorococcus* sp. was about one order of magnitude less abundant than *Synechococcus* sp. and not affected by OA (Supplementary Figure 2 and Table 2). The average abundance of small picoeukaryotes in WINTER (1.1 ± 0.1 × 10^4^ ml^−1^) doubled that observed in SUMMER (0.6 ± 0.1 × 10^4^ ml^−1^, Kruskal–Wallis, *n* = 143, *P* < 0.01) and increased significantly with nutrient additions in both experiments (Figures 1G,H and Table 2). The abundance of large picoeukaryotes was not significantly different between experiments (4.7 ± 4.4 × 10^3^ and 3.4 ± 2.5 × 10^3^ ml^−1^ in WINTER and SUMMER, respectively), increased with nutrient addition, and was stimulated by the lowered pH only in SUMMER (Figures 1I,J and Tables 1, 2).

### Heterotrophic Bacterial Abundance and Activity

**Figure 2:**
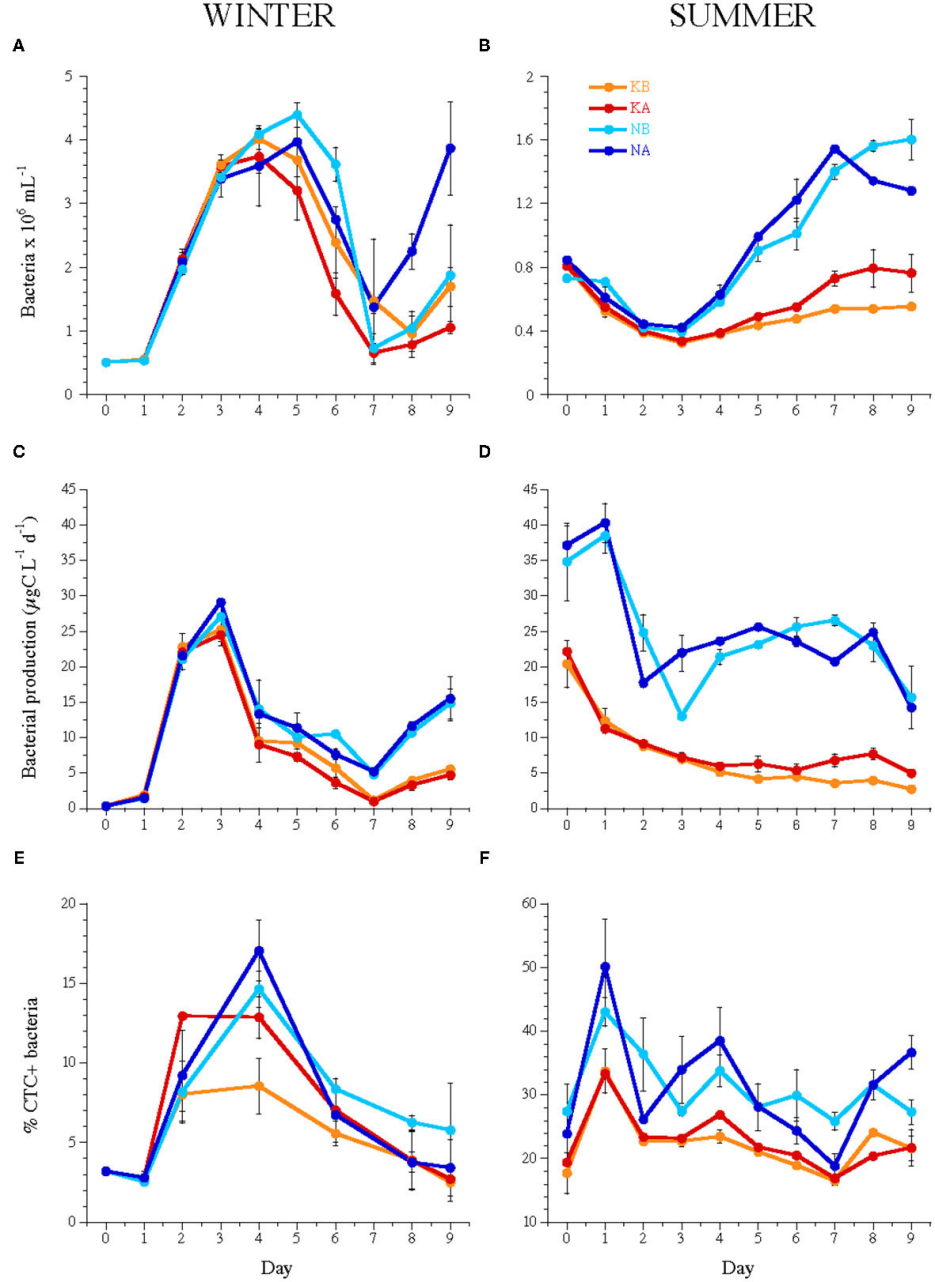
Temporal dynamics of **(A,B)** bacterial abundance, **(C,D)** bacterial heterotrophic production, and **(E,F)** the percentage of CTC-labeled cells, i.e., actively respiring bacteria during the experiments WINTER (left) and SUMMER (right). Bars indicate the maximum and minimum values of experimental duplicates; when not visible, they are contained within the symbol. Note the differences in the y-axis scales of bacterial abundance and % CTC+ cells between seasons. For treatment codes, see Figure 1.

Time-integrated BA was significantly higher in the WINTER (2.3 ± 0.1 × 10^6^ ml^−1^) than in the SUMMER experiment (0.7 ± 0.1 × 10^6^ ml^−1^, Kruskal–Wallis, *n* = 144, *P* < 0.0001), and BA dynamics differed between seasons: in WINTER, BA increased 7.6 times from day 0 until day 4 without differences between treatments (Figure 2A), while in SUMMER, BA decreased initially and increased from day 3 in all treatments but more significantly in the nutrient-amended ones. An acidification effect was found only for the treatments without nutrient addition (Figure 2B and Table 2).

Heterotrophic bacterial carbon production rates increased in WINTER from 0.26 μg C L^−1^ day^−1^ at day 0 to 24.44–29.09 μg C L^−1^ day^−1^ until day 3, while they decreased in SUMMER from initial 21.28 ± 2.91 μg C L^−1^ day^−1^ and 36.01 ± 5.18 μg C L^−1^ day^−1^ in the nutrient unamended and amended treatments, respectively, until days 2–3 (Figures 2C,D). BP was stimulated by nutrient addition but was not affected by lowered pH (Table 2) and was significantly related to the abundance of HNA bacteria except in the SUMMER treatments, where it was related to that of LNA bacteria (Supplementary Table 1). The percentage of CTC+ cells was significantly stimulated by acidification only in the K treatments of WINTER (ANCOVA, *n* = 40, *P* < 0.05, Figures 2E,F).

### Abundance of the Viral Populations

**Figure 3:**
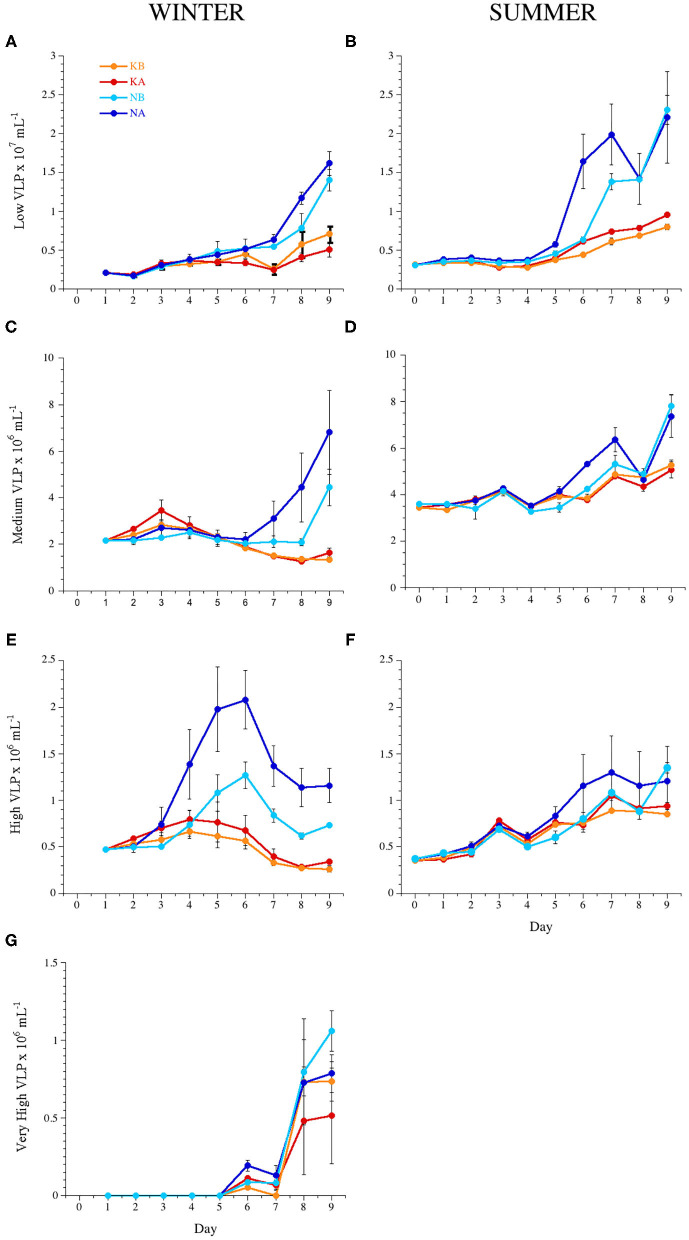
Temporal dynamics of the abundance of the different virus-like particle (VLP) subpopulations: **(A,B)** low, **(C,D)** medium, **(E,F)** high, and **(G)** very high fluorescence VLP during experiments WINTER (left) and SUMMER (right). For treatment codes, see Figure 1.

Total VLP abundance increased after an initial stable phase from day 7 and 5 in experiments WINTER and SUMMER, respectively, and the same behavior was exhibited by the low subpopulation (Figures 3A,B); this increase was significantly higher in the N treatments than in the K treatments (Table 2). Total VLP abundance and the abundance of the MEDIUM subpopulation integrated over the experimental time were significantly lower in the WINTER than in the SUMMER experiment (Figures 3C,D, Kruskal–Wallis, *n* = 144, *P* < 0.0001).

In experiment WINTER, only the high subpopulation was stimulated by acidification (Figures 3E,F, Table 2). A cytometric group of very high fluorescence VLP (very high, Supplementary Figure 1) emerged in the second half of the WINTER experiment (Figure 3G). The abundance of the very high subpopulation was significantly stimulated by nutrient additions but significantly lower in the acidified treatments toward the end of the experiment (ANCOVA, *n* = 24, *P* < 0.05). Total VLP and the subpopulation of low fluorescence VLP, i.e., mostly bacteriophages, were negatively affected by lowered pH with nutrient addition (Table 3).

In the nutrient-deplete SUMMER experiment, in turn, total VLP abundance and the abundances of all subpopulations were correlated negatively to the measured pH in the mesocosms (Table 3), i.e., increased with acidification. For the growth phase of VLP (days 6–9), the lowered pH stimulated the low fluorescence subpopulation, i.e., the bacteriophages, regardless the nutrient addition (Figure 3B, ANCOVA, *n* = 15, *P* < 0.0001). In the nutrient treatments, the medium fluorescence subpopulation abundances, also comprising bacteriophages, were triggered in the acidified containers for the time interval 5–7 days (Figure 3D, ANCOVA, *P* = 0.0011).

### Viral Production and Lysogeny

**Figure 4:**
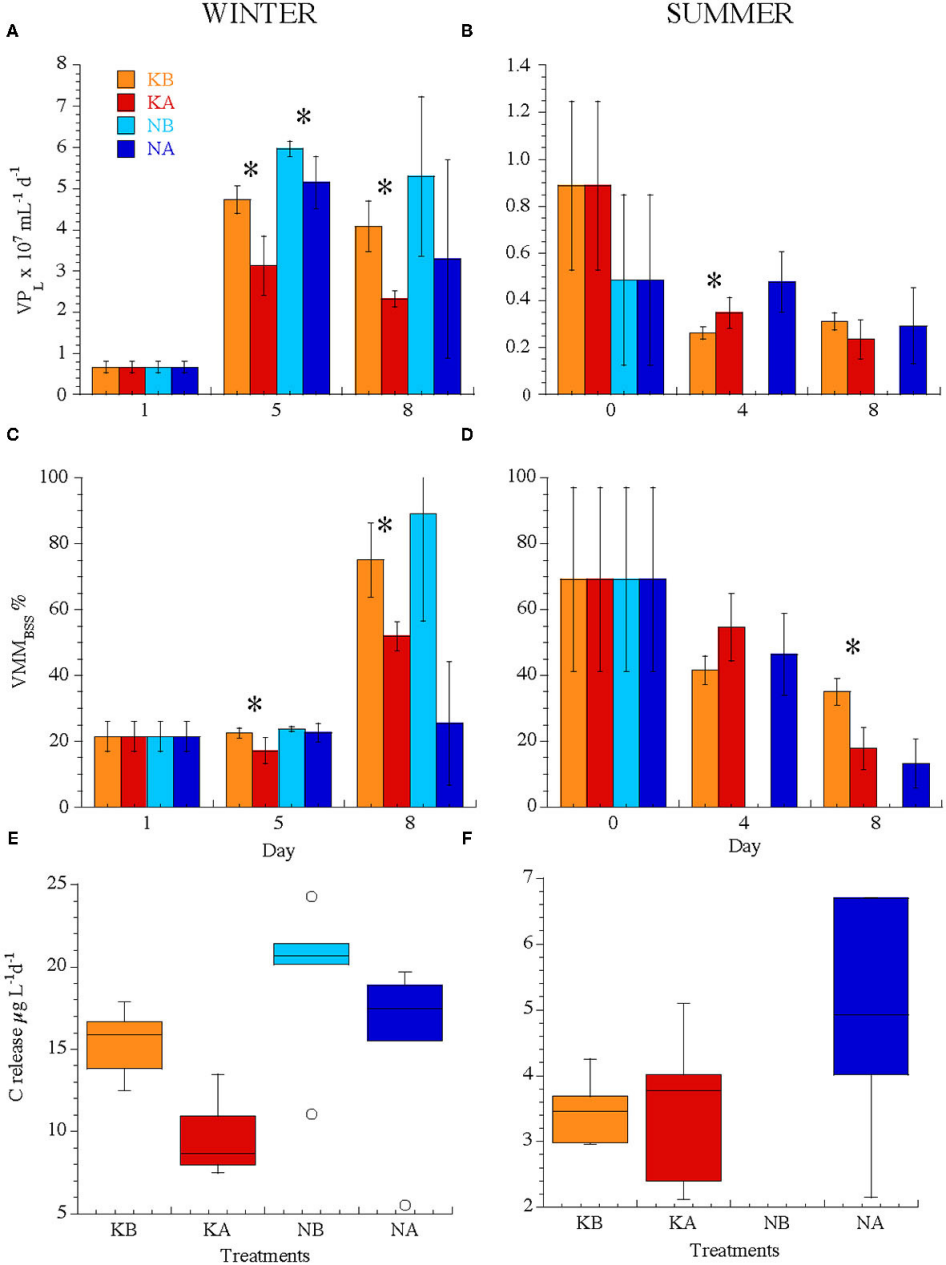
**(A,B)** Lytic viral production (VP_L_), **(C,D)** viral-mediated mortality expressed as a percentage of the bacterial standing stock (VMM_BSS_), **(E,F)** carbon release rates (μg C L^−1^ day^−1^) through viral lysis calculated from VP from all treatments in the experiments WINTER (left) and SUMMER (right). Note the difference in the y-axis scales between seasons, in particular for VP. Bars indicate standard deviations. Stars indicate significant differences between treatments (*P* < 0.05). For treatment codes, see Figure 1.

Initial lytic viral production rates (VP_L_) did not differ significantly between seasons and averaged 0.68 ± 0.32 × 10^7^ VLP ml^−1^ day^−1^ but evolved differently between experiments (Figures 4A,B). In WINTER, VP_L_ increased in all treatments but were significantly lower in the KA treatments (2.73 ± 0.65 × 10^7^ VLP ml^−1^ day^−1^) with respect to KB (4.41 ± 0.56 × 10^7^ VLP ml^−1^ day^−1^, pooled data for days 5 and 8, Kruskal–Wallis, *n* = 12, *P* < 0.01). VP_L_ was significantly stimulated by nutrient addition (Kruskal–Wallis, *n* = 23, *P* < 0.05) with a trend of reduced VP_L_ due to lowered pH (4.41 ± 1.64 × 10^7^ and 5.63 ± 1.13 × 10^7^ VLP ml^−1^ day^−1^ in NA and NB treatments, respectively, Kruskal–Wallis, *n* = 11, *P* = 0.05). Lysogenic production could only be induced at day 0 (0.69 ± 0.01 × 10^7^ VLP ml^−1^ day^−1^) and in the NA treatments at day 5 (3.46 ± 2.96 × 10^7^ VLP ml^−1^ day^−1^, Table 4).

**Table 4:** The fraction of lysogenic viral production (VP_I_) over total viral production, the fraction of infected cells (FIC), and the fraction of lysogenic cells (FLC) in the different treatments from the WINTER and SUMMER experiments.

	**VP_I_ (%)**			**FIC (%)**			**FLC (%)**		
**WINTER**	**Day 1**	**Day 5**	**Day 8**	**Day 1**	**Day 5**	**Day 8**	**Day 1**	**Day 5**	**Day 8**
KB	51	*nd*	*nd*	3.4 ± 1.5	6.2 ± 0.5	53.2 ± 8.6	6.6 ± 0.3	*nd*	*nd*
KA	51	*nd*	*nd*	3.4 ± 1.5	3.6 ± 0.9	40.3 ± 4.1	6.6 ± 0.3	*nd*	*nd*
NB	51	*nd*	*nd*	3.4 ± 1.5	3.3 ± 1.9	7.4 ± 2.7	6.6 ± 0.3	*nd*	*nd*
NA	51	40	*nd*	3.4 ± 1.5	1.8 ± 0.9	2.1 ± 1.7	6.6 ± 0.3	0.9 ± 0.4	*nd*
**SUMMER**	**Day 0**	**Day 4**	**Day 8**	**Day 0**	**Day 4**	**Day 8**	**Day 0**	**Day 4**	**Day 8**
KB	27	*nd*	*nd*	7.5 ± 4.8	3.4 ± 1.5	6.6 ± 3.6	7.0 ± 0.8	*nd*	*nd*
KA	27	35	*nd*	7.5 ± 4.8	6.9 ± 2.7	2.6 ± 0.9	7.0 ± 0.8	6.0 ± 2.0	*nd*
NB	*nd*	100	100	2.0 ± 1.1	*nd*	*nd*	2.7 ± 0.3	4.5 ± 1.6	3.6 ± 2.2
NA	*nd*	*nd*	*nd*	2.0 ± 1.1	7.0 ± 1.8	1.5 ± 0.8	2.7 ± 0.3	*nd*	*nd*

*FIC and FLC values are averages ± standard deviations from triplicates*.

*nd, not detected*.

In the SUMMER experiment, initial VP_L_ rates decreased in the K treatments on days 4 and 8 (Figure 4B, Kruskal–Wallis, *n* = 15, *P* < 0.05) and were not affected by lowered pH. In the NB treatments, lysogenic production rates accounted for 100% of virus production, i.e., virus production could only be detected in the treatments where lysogens were induced; VP_I_ were 0.30 ± 0.11 × 10^7^ and 0.48 ± 0.13 × 10^7^ VLP ml^−1^ day^−1^ at days 4 and 8, respectively, being lower or equal compared to the VP_L_ rates in the acidified, i.e., NA, treatments. Moreover, VP_I_ could only be induced at day 0 without nutrient addition (0.33 ± 0.11 × 10^7^ VLP ml^−1^ day^−1^) and at day 4 in the KA treatments (0.19 ± 0.14 × 10^7^ VLP ml^−1^ day^−1^, Table 4).

Calculated BS averaged 57 ± 50 in WINTER (15–226) and 16 ± 10 (4–43) in SUMMER. The fraction of infected bacteria (FIC) was low in both experiments and all treatments and accounted for 13.9 ± 18.9% in WINTER and 4.8 ± 3.2% in SUMMER. Only in WINTER, FIC decreased with nutrient additions (Kruskal–Wallis, *n* = 23, *P* < 0.05) and was not affected by acidification. The FLC also remained low throughout the experiments and treatments without discernable treatment effects (Table 4).

### Viral-Mediated Bacterial Mortality and Carbon Fluxes

The viral-mediated loss of bacterial standing stock (VMM_BSS_ day^−1^) did not differ between seasons (39 ± 28% in WINTER and 41 ± 22% in SUMMER) but was initially lower in WINTER (21 ± 5%) than in SUMMER (54 ± 30%, Figures 4C,D). VMM_BSS_ was lowered by acidification only in WINTER (Figures 4C,D, Kruskal–Wallis, *n* = 23, *P* < 0.05). Carbon released from the particulate to the dissolved pool due to viral activity was four times higher in WINTER (15.0 ± 5.2 μg C L^−1^ day^−1^) than in SUMMER (3.9 ± 1.3 μg C L^−1^ day^−1^, Kruskal–Wallis, *n* = 40, *P* < 0.0001, Figures 4E,F). Only in WINTER viral-mediated carbon flux was significantly and negatively affected by acidification (Figure 4E, Kruskal–Wallis, *n* = 23, *P* < 0.05) and also stimulated by nutrient addition (Figure 4, Kruskal–Wallis, *n* = 23, *P* < 0.01).

After pooling all data from both seasons and all treatments, viral-mediated carbon flux increased with pH (Spearman ρ = 0.56, *P* = 0.0002, *n* = 40) and was positively correlated to chl *a* concentration and BA (Spearman ρ = 0.67 and 0.75, respectively, *P* < 0.0001, *n* = 40). However, when separating by seasons and nutrient treatments, the trend of increasing viral production and viral-mediated mortality with increasing pH was significant only in the K treatments of the WINTER experiment (Table 3). In addition, lytic viral production correlated significantly and positively with bacterial abundance and production only in WINTER without nutrient amendment (Supplementary Tables 1, 2).

## Discussion

*Ex situ* mesocosm experiments were performed to study the effect of acidification and eutrophication on virioplankton and viral activity under controlled laboratory conditions. Although controlled *ex situ* experimental conditions may be biased by avoiding processes that could have intervened in the natural environment, they allow to identify potential mechanisms in the response of the viral community to the studied anthropogenic stressors.

The addition of nutrients stimulated viral production regardless of the season or initial conditions. While in the nutrient-replete situation of the WINTER experiment, the abundance of viral populations and bacteriophage production tended to be negatively affected by OA; in the SUMMER experiment, we found a stimulating trend of lowered pH on viral abundance and production. Nutrient availability seems to play a major role in modulating the effect of increasing *p*CO_2_ on viruses, but the composition and physiological state of the initial plankton communities also modified OA effects on viral-mediated mortality and on the resulting carbon fluxes.

### Effect of OA and Eutrophication on Phytoplankton Viruses

In the WINTER experiment, we found a population of very high fluorescence VLP, as identified by their signature in the cytometric plot of high side scatter and fluorescence, comparable to the cytometric signature of viruses infecting small haptophytes and chlorophytes (Brussaard, 2004). The fact that this population was emerging in the decline phase of the bloom of the “large picoeukaryotes” cytometric group, mostly constituted of small mixotrophic haptophytes (2–4 μm, Massana, pers. comm.), which are the most abundant nanoflagellates at the sampling site year-round (Unrein et al., 2014), suggests that these were actually their potential viruses. Very high fluorescence VLP could not be detected in the SUMMER experiment, where large picoeukaryotes were less abundant. The abundance of these VLP tended to be lower at lowered pH although their putative hosts' abundance was enhanced. Consistently, Larsen et al. (2008) found a decreased production of nanophytoplankton-specific viruses (e.g., infecting *Emiliania huxleyi*) coupled to increased host production under elevated *p*CO_2_, suggesting that lower viral production was not a secondary effect of reduced host production but a direct and negative effect on viral–host interactions. Maat et al. (2014) found viral lysis of *M. pulsilla* reduced under enhanced *p*CO_2_ and P-limited conditions. In addition, during an acidification experiment in the Arctic, very small sized picophytoplankton was stimulated by elevated levels of *p*CO_2_, while the viral group associated to the decline in the picophytoplankton bloom and apparently responsible for that decline was not found to be affected by *p*CO_2_ (Brussaard et al., 2013).

In WINTER, cyanobacteria of the genus *Synechococcus* were negatively affected by OA, while the subpopulation of high fluorescence VLP was stimulated regardless of the nutrient conditions (Table 2 and Figure 3E). The high subpopulation is generally constituted by cyanophages and viruses of picoeukaryotes (Brussaard, 2004; Brussaard et al., 2008a; Martinez et al., 2014). The positive correlation of this subpopulation to *Synechococcus* (Spearman ρ = 0.57, *n* = 72, *P* < 0.0001) and to *Prochlorococcus* (Spearman ρ = 0.41, *n* = 72, *P* = 0.0004) lends further support that this group was mainly comprised of cyanophages. Thus, the negative response of *Synechococcus* in WINTER could be the consequence of enhanced viral lysis in this season. Indeed, freshwater cyanophages have been shown to tolerate a broad range of pH (Suttle, 2000). Moreover, in the SUMMER experiment, where the pH changed over 0.6 U, these putative cyanophages were stimulated by acidification in both nutrient treatments (Table 3). There, however, *Synechococcus* was stimulated in the OA treatments, at least from day 5 in the nutrient control treatments, where pH reached the lowest values due to microbial activities (Figure 1). Several studies suggest a stimulating effect of increased *p*CO_2_ on cyanobacteria production due to energy savings related to the reduced use of carbon concentration mechanisms (Fu et al., 2007; Raven et al., 2012). In the case of the oligotrophic conditions of the SUMMER experiment, the enhanced production of putative cyanophages might appear as a secondary effect of enhanced host production under elevated levels of *p*CO_2_. In fact, studies with the model system cyanophage S-PM2 and its host *Synechococcus* sp. WH7803 revealed a negative effect of OA on the growth rate of *Synecchococcus*, which compromised biogenesis and the replication cycle of S-PM2 (Traving et al., 2013).

### Effect of OA and Eutrophication on Bacteriophage Production

In WINTER, bacterial production increased during the first day, when bacteriophage production was significantly reduced under OA, and this was most apparent in the nutrient control treatments. Since bacterial host abundance was stimulated or not affected by pH in the nutrient amended and control treatments, respectively (Table 3), phage replication rates or phage–host interactions seem to be directly affected by acidification. In fact, metatranscriptome analysis from WINTER found that phage encoding genes most similar to *Podoviridae* phages infecting SAR11-clade bacteria were negatively affected by acidification, and this was more conspicuous in the non-amended treatments (Bunse et al., 2016).

If bacterial species resistant to viral infection are promoted by ocean acidification, this could affect phage production and total bacterial mortality. High throughput sequencing of 16S ribosomal RNA (rRNA) gen amplicons from the present study showed the synergistic effects of OA and nutrient amendment selected for some specific OTUs belonging to SAR86 (Baltar et al., 2015). However, SAR86 OTUs had only a minor contribution to total bacterial community and might not have been responsible for the reduction in phage-mediated bacterial mortality in the acidification treatments of WINTER (Figure 4C). Moderate changes in pH were shown to cause compositional shifts in the bacterial community from the North Sea in all seasons (Krause et al., 2012). Conversely, most other OA mesocosm experiments showed a lack of clear *p*CO_2_ effects on global bacterial community structure (Newbold et al., 2012; Roy et al., 2013; Sperling et al., 2013; Zhang et al., 2013; Oliver et al., 2014), and thus, changes in the bulk bacterial mortality are unlikely caused by the selection of lysis-resistant bacterial species through acidification.

Instead of looking at slight phylogenetic changes in the bacterial community as a response to OA, the metabolic state of the bacterial hosts, which is crucial for successful viral infection, proliferation, and bulk viral production, should be considered. Metatranscriptome analyses from the WINTER experiment showed that bacteria adjusted their gene expression patterns to acidification by enhancing the expression of genes encoding proton pumps, such as respiration complexes, proteorhodopsin, and membrane transporters in the nutrient control treatments (Bunse et al., 2016). Proton transport across membranes demands much energy at the expense of bacterial growth and growth efficiency. Since viral infection and BS rely on the bacterial growth rate and growth efficiency (Middelboe, 2000), viral production rates should decrease with lower bacterial growth efficiency. Actually, VP rates were significantly and positively correlated to bacterial heterotrophic production and abundance in the nutrient deplete controls (Supplementary Figure 1). There, the lowered pH stimulated the percentage of CTC-labeled cells but did not affect bacterial heterotrophic production rates (Figure 2), suggesting lower bacterial growth efficiency under OA as demonstrated from previous experiments (Motegi et al., 2013; Celussi et al., 2017) with implications for bacteriophage production.

Concerning lysogeny as a potential viral strategy to cope with unfavorable conditions (Weinbauer, 2004), we found a very low incidence of the lysogenic life style with no discernible effect of OA (Table 4) in accordance with a previous study from the Mediterranean (Tsiola et al., 2017). Excluding day 0, only 4 out of 12 measurements of viral production showed lysogenic production induced by mitomycin C (MC). Microcosm experiments to discern the effect of the viral life style on bacterial community composition have shown consistently lower viral and bacterial numbers in the MC treatments (Chen et al., 2019), suggesting harmful effects of the inducing reagent itself on bacterial growth, inhibiting certain bacterial clades (Hewson and Fuhrman, 2007). However, in the NB treatments of SUMMER, temperate viruses accounted for the totality of viral production. It has been suggested that lysogeny dominates in oligotrophic environments with low host abundances and contact rates (Weinbauer et al., 2003a) and that prophage induction occurs in rapidly growing cells (Wilson and Mann, 1997). Moreover, protistan grazing also increases bacterial growth rates by recycling nutrients (Simek et al., 2001; Weinbauer et al., 2003b), and this might induce lysogens into the lytic cycle. Unfortunately, grazing rates were not measured in the present study, but the bacterial dynamics and total phytoplankton biomass in SUMMER were very similar to a previous experiment from the same sampling site, where grazing accounted for up to 100% of the bacterial standing stock (Baltar et al., 2016). Interestingly, and similar to the present study, VLP reached considerably high numbers at the end of that experiment (Sandaa et al., 2009).

### Modulation of OA Effects on the Microbial Community by Seasonality and Nutrient Availability

The response of the microbial community and their associated viruses to lowered pH differed considerably between seasons. Higher *p*CO_2_ did not stimulate auto- and heterotrophic production of the microbial community in a nutrient replete situation in WINTER, while it did stimulate the microbial community from an oligotrophic situation in SUMMER (Figures 1, 2; Sala et al., 2016). Besides the nutrient availability, the seasonal differences were most conspicuous in the different successional stages of the plankton community with a diatom-dominated phytoplankton bloom in winter and pico- and nanoflagellates prevailing in the summer plankton community (Sala et al., 2016).

In our experiments, total VLP abundance and the abundance of VLP subpopulations were intimately linked to microbial production dynamics, in agreement with Larsen et al. (2001, 2004). They were also stimulated consistently by acidification in both nutrient treatments in SUMMER, as a consequence of the cascading effect of OA on phytoplankton and bacterial production in the oligotrophic situation (Table 3). This finding contrasted with the nutrient-replete WINTER situation (Figure 4), where phage-mediated processes were significantly reduced by OA. These differences in the pH sensitivity of viral production and host production is in accordance with results from the Pelagic Ecosystem CO_2_ Enrichment studies (PeECE I–III). There, bacterial production was either not affected (Allgaier et al., 2008) or stimulated by lowered pH (Grossart et al., 2006). However, these experiments were performed under similar conditions, same site and approximately the same dates, but under different trophic conditions. This was translated into a lower total phytoplankton biomass. Riebesell et al. (2008) argued that the trophic state of the system, i.e., limitation by inorganic and organic nutrients, should influence, at least indirectly, the microbial community.

Indeed, the effects of lowered pH on viral abundance or phage-mediated bacterial mortality were more pronounced after the chlorophyll peak. Significant effects of acidification on the microbial community have been found to occur after the bloom phase (Arnosti et al., 2011; Sperling et al., 2013). During an *in situ* mesocosm experiment, elevated levels of *p*CO_2_ triggered higher diversity of the particle-associated bacterial community only after the breakdown of the phytoplankton bloom (Sperling et al., 2013) concomitant with increased dissolved and particulate primary production (Engel et al., 2013) and bacterial production (Piontek et al., 2013), but with decreased bacterial abundance (Brussaard et al., 2013). The latter was suggested to be due to enhanced viral lysis rates in a high *p*CO_2_ postbloom situation.

### Implications for the Carbon Fluxes in the Microbial Food Web

In the experiments performed with a nano–picoplankton-dominated community, in the SUMMER oligotrophic situation, phage-mediated bacterial mortality was, in general, low without a clear effect of OA. Nonetheless, phage abundances were high. A 3-year study from the same sampling site revealed that a specific class of organic aggregates, the transparent exopolymeric particles (TEP), recurrently accumulate in the stratified, oligotrophic conditions of early summer (Ortega-Retuerta et al., 2018). Suspended organic aggregates are hotspots for bacterial growth (Azam and Long, 2001; Azam and Malfatti, 2007), and high viral abundance found on TEP (Mari et al., 2007) suggests high viral infection of TEP-attached bacteria. It must be pointed out that the virus production assays (VRAs) used in the present study exclude aggregate-attached bacteria due to prefiltration of the water and, consequently, phage production associated to TEPs. This might explain the discrepancy between the low phage production and high viral numbers observed. However, a simulation model of a *Phaeocystis globosa* bloom demonstrated that TEPs scavenge viral particles and reduce their infectivity (Ruardij et al., 2005). Moreover, infected host cells might be grazed selectively, lowering the contact rate between viruses and uninfected host cells (Ruardij et al., 2005). In fact, seasonal studies from the same sampling site show peaks of nanoflagellate abundance and relatively important grazing rates in summer (Boras et al., 2009; Unrein et al., 2014) suggesting a less efficient viral shunt in that season.

Instead, phage-mediated loss of bacterial biomass in WINTER was apparently the dominant mortality agent for bacteria there. Since bacterial abundance was higher in WINTER than in SUMMER, this may have increased the encounter probability with the host. In addition, it has been suggested that viruses are the prevailing mortality agent in eutrophic waters over protistan grazing (Steward et al., 1996), consistent with observations in nutrient-rich bloom/postbloom situations where the organic carbon in the microbial food web is mainly processed by the viral shunt (Malits et al., 2014). Viral lysis, by destroying the infected cell and releasing the content of the cytoplasm to the environment, mainly proteins and glucose (Boras et al., 2015), feeds the DOC pool and reduces the transfer of photosynthetically fixed carbon to higher trophic levels and to export; hence, retaining it in the basal levels of the planktonic food web (Sheik et al., 2014), increasing bacterial respiration (Middelboe and Lyck, 2002; Bonilla-Findji et al., 2008), and weakening the biological carbon pump, i.e., the flux of organic carbon to the deep ocean.

We found phage production and concomitant organic carbon release rates significantly reduced in the acidified treatments of the nutrient replete WINTER experiment, especially in the non-amended treatments. When comparing the organic carbon processed by the viral shunt with the total autotrophic biomass converted to organic carbon with a C/Chl *a* ratio estimated for bloom conditions in the Northwest Mediterranean (Gutiérrez-Rodríguez et al., 2010), viral lysis transferred 41 ± 5% of the phytoplankton standing stock to the DOC pool, compared to 18 ± 8% under acidification. Although carbon release rates calculated from VP might be overestimated due to the plausible stimulation of bacterial production in the VRA experiments (Weinbauer et al., 2009), it is evident that OA reduces the DOC supply for bacterial activity mediated by viral lysis (Malits and Weinbauer, 2009).

While no significant differences in the net accumulation of DOC and the protein-like DOM compounds could be detected between the acidified and non-acidified conditions in the present study (Aparicio et al., 2016), enhanced DOC production under elevated *p*CO_2_ concentrations has often been observed in published studies (Kim et al., 2011; Yoshimura et al., 2013; Paul et al., 2015). Mesocosm experiments in the Arctic Ocean have shown that DOC exudation by phytoplankton increased under acidification but without concomitant net DOC accumulation (Engel et al., 2013), suggesting that the increased bacterial biomass production (Piontek et al., 2013) was responsible for the DOC removal. Since bacterial heterotrophic production was not stimulated under OA in WINTER, reduced DOC release mediated by viral lysis under acidification could have been compensated by increased DOC production under lowered pH conditions (Kim et al., 2011; Engel et al., 2013; Yoshimura et al., 2013; Paul et al., 2015). This would have led to non-significant differences in net DOC accumulation between treatments, as we observed (Aparicio et al., 2016).

Seawater culture experiments with bacterioplankton communities from contrasting sites such as the coastal upwelling influenced Santa Barbara Channel, and the oligotrophic systems of the Sargasso Sea and the South Pacific Subtropical Gyre evidenced that elevated *p*CO_2_ led to greater DOC removal by bacteria growing on a range of added DOC types, from glucose to complex phytoplankton products (James et al., 2017). The authors inferred higher bacterioplankton respiration rates under OA from changes in DOC and bacterial biomass and suggested reduced vertical DOC export. In the present study, we indeed found that actively respiring bacterial cells labeled by CTC increased with decreasing pH, at least in the nutrient amended treatments (Table 3), in accordance with previous studies in the Mediterranean (Celussi et al., 2017). Together with the lower calcification rates of planktonic organisms and a consequently reduced particle flux to the deep ocean in an acidified environment (Engel et al., 2005), this results in a positive feedback to atmospheric *p*CO_2_. Reduced viral lysis under OA, however, should counterbalance these negative effects of lowered pH on the biological carbon pump, at least in bloom and postbloom situations where viral lysis plays a relevant role in carbon cycling through the microbial food web.

## Conclusions

Our experimental results provide insight into how two major global change pressures, ocean acidification and eutrophication, modulate viral production and viral-mediated processes in the plankton community at different seasons. While nutrient loading consistently enhanced phage production regardless of the season, the effect of OA on viruses differed between seasons. In the nutrient-replete bloom/postbloom situation in WINTER, viral production and phage-mediated bacterial mortality were reduced under increased *p*CO_2_. In the oligotrophic SUMMER situation, where viral-mediated carbon fluxes were less important compared to WINTER, OA stimulated viruses through the cascading effect of elevated *p*CO_2_ on the autotrophic and heterotrophic production. Given the relevant role of viruses in marine carbon cycling and the biological carbon pump, these two anthropogenic stressors may modulate carbon fluxes through their effect on viruses at the base of the pelagic food web in future global change scenarios.

## Originality—Significance Statement

This is the first study that shows phage-mediated bacterial mortality leading to altered carbon fluxes in response to elevated *p*CO2 and nutrient additions simulating the combined effect of acidification and eutrophication. This work contributes to the understanding of how these two anthropogenic stressors modulate carbon fluxes at the base of the pelagic food web in future global change scenarios.

## Data Availability Statement

The original contributions presented in the study are included in the article/Supplementary Materials, further inquiries can be directed to the corresponding authors.

## Author Contributions

AM, JB, VB, EC, CP, JP, JG, CM, MS, and DV conceived, planned, and carried out the mesocosm experiments. JB and AM performed the mortality experiments. AM analyzed viruses by flow cytometry and took the lead in writing the manuscript. All authors provided critical feedback and helped to shape the manuscript.

## Conflict of Interest

The authors declare that the research was conducted in the absence of any commercial or financial relationships that could be construed as a potential conflict of interest.

## References

[B1] Allgaier M. Riebesell U. Vogt M. Thyrhaug R. Grossart H. P. (2008). Coupling of heterotrophic bacteria to phytoplankton bloom development at different *p*CO_2_ levels: a mesocosm study. Biogeosciences 5, 1007–1022. 10.5194/bg-5-1007-2008

[B2] Alonso-Sáez L. Balagué V. Sà E. L. Sánchez O. González J. M. Pinhassi J.. (2007). Seasonality in bacterial diversity in north-west Mediterranean coastal waters: assessment through clone libraries, fingerprinting and FISH. FEMS Microbiol. Ecol. 60, 98–112. 10.1111/j.1574-6941.2006.00276.x 17250750

[B3] Aparicio F. L. Nieto-Cid M. Borrull E. Calvo E. Pelejero C. Sala M. M.. (2016). Eutrophication and acidification: do they induce changes in the dissolved organic matter dynamics in the coastal Mediterranean Sea? Sci. Total Environ. 563–564, 179–189. 10.1016/j.scitotenv.2016.04.108 27135581

[B4] Arnosti C. Grossart H. P. Mhling M. Joint I. Passow U. (2011). Dynamics of extracellular enzyme activities in seawater under changed atmospheric pCO_2_: a mesocosm investigation. Aquat. Microb. Ecol. 64, 285–298. 10.3354/ame01522

[B5] Azam F. (1998). Microbial control of oceanic carbon flux: the plot thickens. Science 280, 694–696. 10.1126/science.280.5364.694

[B6] Azam F. Fenchel T. Field J. G. Gray J. S. Meyer-Reil L. A. Thingstad F. (1983). The ecological role of water-column microbes in the sea. Mar. Ecol. Prog. Ser. 10, 257–263. 10.3354/meps010257

[B7] Azam F. Long R. A. (2001). Sea snow microcosms. Nature 414, 495,497–498. 10.1038/35107174 11734832

[B8] Azam F. Malfatti F. (2007). Microbial structuring of marine ecosystems. Nat. Rev. Micro 5, 782–791. 10.1038/nrmicro1747 17853906

[B9] Baltar F. Palovaara J. Unrein F. Catala P. Hornák K. Šimek K.. (2016). Marine bacterial community structure resilience to changes in protist predation under phytoplankton bloom conditions. ISME J. 10, 568–581. 10.1038/ismej.2015.135 26262814 PMC4817682

[B10] Baltar F. Palovaara J. Vila-Costa M. Salazar G. Calvo E. Pelejero C.. (2015). Response of rare, common and abundant bacterioplankton to anthropogenic perturbations in a Mediterranean coastal site. FEMS Microbiol. Ecol. 91:fiv058. 10.1093/femsec/fiv058 26032602

[B11] Berdjeb L. Pollet T. Domaizon I. Jacquet S. (2011). Effect of grazers and viruses on bacterial community structure and production in two contrasting trophic lakes. BMC Microbiol. 11:88. 10.1186/1471-2180-11-88 21527043 PMC3114703

[B12] Bonilla-Findji O. Malits A. Lefèvre D. Rochelle-Newall E. Lemée R. Weinbauer M. G.. (2008). Viral effects on bacterial respiration, production and growth efficiency: consistent trends in the Southern Ocean and the Mediterranean Sea. Deep. Res. Part II Top. Stud. Oceanogr. 55, 559–912. 10.1016/j.dsr2.2007.12.004

[B13] Bopp L. Resplandy L. Orr J. C. Doney S. C. Dunne J. P. Gehlen M.. (2013). Multiple stressors of ocean ecosystems in the 21st century: projections with CMIP5 models. Biogeosciences 10, 6225–6245. 10.5194/bg-10-6225-2013

[B14] Boras J. A. Sala M. M. Vázquez-Domínguez E. Weinbauer M. G. Vaqué D. (2009). Annual changes of bacterial mortality due to viruses and protists in an oligotrophic coastal environment (NW Mediterranean). Env. Microbiol. 11, 1181–1193. 10.1111/j.1462-2920.2008.01849.x 19207563

[B15] Boras J. A. Vaqué D. Maynou F. Sà E. L. Weinbauer M. G. Sala M. M. (2015). Factors shaping bacterial phylogenetic and functional diversity in coastal waters of the NW Mediterranean Sea. Estuar. Coast. Shelf Sci. 154, 102–110. 10.1016/j.ecss.2014.12.039

[B16] Borges A. V. Gypens N. (2010). Carbonate chemistry in the coastal zone responds more strongly to eutrophication than ocean acidification. Limnol. Oceanogr. 55, 346–353. 10.4319/lo.2010.55.1.0346

[B17] Brussaard C. P. D. (2004). Optimization of procedures for counting viruses by flow cytometry. Appl. Environ. Microbiol. 70, 1506–1513. 10.1128/AEM.70.3.1506-1513.2004 15006772 PMC368280

[B18] Brussaard C. P. D. (2009). Enumeration of bacteriophages using flow cytometry, in Bacteriophages: Methods and Protocols, Vol. 1: Isolation, Characterization, and Interactions, eds Clokie M. R. J. Kropinski A. M. (Totowa, NJ: Humana Press), 97–111. 10.1007/978-1-60327-164-6_11 19066815

[B19] Brussaard C. P. D. Noordeloos A. A. M. Witte H. Collenteur M. C. J. Schulz K. Ludwig A.. (2013). Arctic microbial community dynamics influenced by elevated CO_2_ levels. Biogeosciences 10, 719–731. 10.5194/bg-10-719-2013

[B20] Brussaard C. P. D. Timmermans K. R. Uitz J. Veldhuis M. J. W. (2008a). Virioplankton dynamics and virally induced phytoplankton lysis versus microzooplankton grazing southeast of the Kerguelen (Southern Ocean). Deep Sea Res. Part II Top. Stud. Oceanogr. 55, 752–765. 10.1016/j.dsr2.2007.12.034

[B21] Brussaard C. P. D. Wilhelm S. W. Thingstad F. Weinbauer M. G. Bratbak G. Heldal M.. (2008b). Global-scale processes with a nanoscale drive: the role of marine viruses. ISME J. 2, 575–578. 10.1038/ismej.2008.31 18385772

[B22] Bunse C. Lundin D. Karlsson C. M. G. Akram N. Vila-Costa M. Palovaara J.. (2016). Response of marine bacterioplankton pH homeostasis gene expression to elevated CO_2_. Nat. Clim. Chang. 6, 483–487. 10.1038/nclimate2914

[B23] Cai W.-J. Hu X. Huang W.-J. Murrell M. C. Lehrter J. C. Lohrenz S. E.. (2011). Acidification of subsurface coastal waters enhanced by eutrophication. Nat. Geosci. 4:766. 10.1038/ngeo1297

[B24] Caldeira K. Wickett M. E. (2003). Oceanography: anthropogenic carbon and ocean pH. Nature 425:365. 10.1038/425365a 14508477

[B25] Celussi M. Malfatti F. Annalisa F. Gazeau F. Giannakourou A. Pitta P.. (2017). Ocean acidification effect on prokaryotic metabolism tested in two diverse trophic regimes in the Mediterranean Sea. Estuar. Coast. Shelf Sci. 186, 125–138. 10.1016/j.ecss.2015.08.015

[B26] Chen X. Ma R. Yang Y. Jiao N. Zhang R. (2019). Viral regulation on bacterial community impacted by lysis-lysogeny switch: a microcosm experiment in eutrophic coastal waters. Front. Microbiol. 10:1763. 10.3389/fmicb.2019.01763 31417537 PMC6685395

[B27] Clayton T. D. Byrne R. H. (1993). Spectrophotometric seawater pH measurements: total hydrogen ion concentration scale calibration of m-cresol purple and at-sea results. Deep Sea Res. Part I Oceanogr. Res. 40, 2115–2129. 10.1016/0967-0637(93)90048-8

[B28] Crawfurd K. J. Alvarez-Fernandez S. Mojica K. D. A. Riebesell U. Brussaard C. P. D. (2017). Alterations in microbial community composition with increasing *f* CO_2_: a mesocosm study in the eastern Baltic Sea. Biogeosciences 14, 3831–3849. 10.5194/bg-14-3831-2017

[B29] Danovaro R. Corinaldesi C. Dell'anno A. Fuhrman J. A. Middelburg J. J. Noble R. T.. (2011). Marine viruses and global climate change. FEMS Microbiol. Rev. 35, 993–1034. 10.1111/j.1574-6976.2010.00258.x 21204862

[B30] Diaz R. J. Rosenberg R. (2008). Spreading dead zones and consequences for marine ecosystems. Science 321, 926–929. 10.1126/science.1156401 18703733

[B31] Dickson A. G. Sabine C. L. Christian J. R. (2007). Guide to Best Practice for Ocean CO_2_ Measurements. PICES Special Publication 3 (Sidney: North Pacific Marine Science Organization).

[B32] Dlugokencky E. Tans P. (2020). Trends in atmospheric carbon dioxide. National Oceanic & Atmospheric Administration, Earth System Research Laboratory (NOAA/ESRL). Available online at: http://www.esrl.noaa.gov/gmd/ccgg/trends/global.html

[B33] Doney S. C. Fabry V. J. Feely R. A. Kleypas J. A. (2009). Ocean acidification: the other CO_2_ problem. Ann. Rev. Mar. Sci. 1, 169–192. 10.1146/annurev.marine.010908.163834 21141034

[B34] Dore J. E. Lukas R. Sadler D. W. Church M. J. Karl D. M. (2009). Physical and biogeochemical modulation of ocean acidification in the central North Pacific. Proc. Natl. Acad. Sci. U.S.A. 106, 12235–12240. 10.1073/pnas.0906044106 19666624 PMC2716384

[B35] Engel A. Borchard C. Piontek J. Schulz K. G. Riebesell U. Bellerby R. (2013). CO_2_ increases 14C primary production in an Arctic plankton community. Biogeosciences 10, 1291–1308. 10.5194/bg-10-1291-2013

[B36] Engel A. Zondervan I. Aerts K. Beaufort L. Benthien A. Chou L.. (2005). Testing the direct effect of CO_2_ concentration on a bloom of the coccolithophorid *Emiliania huxleyi* in mesocosm experiments. Limnol. Oceanogr. 50, 493–507. 10.4319/lo.2005.50.2.0493

[B37] Field C. B. Behrenfeld M. J. Randerson J. T. Falkowski P. (1998). Primary production of the biosphere: integrating terrestrial and oceanic components. Science 281, 237–240. 10.1126/science.281.5374.237 9657713

[B38] Fu F.-X. Warner M. E. Zhang Y. Feng Y. Hutchins D. A. (2007). Effects of increased temperature and CO_2_ on photosynthesis, growth and elemental ratios in marine in marine *Synechococcus* and *Prochlorococcus* (cyanobacteria). J. Phycol. 43, 485–496. 10.1111/j.1529-8817.2007.00355.x

[B39] Fuhrman J. A. (1999). Marine viruses and their biogeochemical and ecological effects. Nature 399, 541–548. 10.1038/21119 10376593

[B40] Galloway J. N. Dentener F. J. Capone D. G. Boyer E. W. Howarth R. W. Seitzinger S. P.. (2004). Nitrogen cycles: past, present, and future. Biogeochemistry 70, 153–226. 10.1007/s10533-004-0370-0

[B41] Gasol J. M. Arístegui J. (2007). Cytometric evidence reconciling the toxicity and usefulness of CTC as a marker of bacterial activity. Aquat. Microb. Ecol. 46, 71–83. 10.3354/ame046071

[B42] Gasol J. M. Cardelús C. Morán X. A. G. Balagué V. Forn I. Marrasé C.. (2016). Seasonal patterns in phytoplankton primary production and photosynthetic parameters in a coastal time-series station of the NW Mediterranean Sea. Scientia Marina, 80S1, 63–77. 10.3989/scimar.04480.06E

[B43] Gasol J. M. Morán X. A. G. (2015). Flow cytometric determination of microbial abundances and its use to obtain indices of community structure and relative activity, in Hydrocarbon and Lipid Microbiology Protocols, eds McGenity T. J. Timmis K. N. Nogales B. (Berlin; Heidelberg: Springer Protocols Handbooks). 10.1007/8623_2015_139

[B44] Gasol J. M. Zweifel U. L. Peters F. Fuhrman J. A. Hågström A. (1999). Significance of size and nucleic acid content heterogeneity as measured by flow cytometry in natural planktonic bacteria. Appl. Env. Microbiol. 65, 4475–4483. 10.1128/AEM.65.10.4475-4483.1999 10508078 PMC91596

[B45] Gobler C. J. Hutchins D. A. Fisher N. S. Cosper E. M. Sanudo-Wilhelmy S. A. (1997). Release and bioavailability of C, N, P, Se., and Fe following viral lysis of a marine chrysophyte. Limnol. Ocean. 42, 1492–1504. 10.4319/lo.1997.42.7.1492

[B46] Grossart H. P. Allgaier M. Passow U. Riebesell U. (2006). Testing the effect of CO_2_ concentration on the dynamics of marine heterotrophic bacterioplankton. Limnol. Ocean. 51, 1–11. 10.4319/lo.2006.51.1.0001

[B47] Gutiérrez-Rodríguez A. Latasa M. Estrada M. Vidal M. Marrasé C. (2010). Carbon fluxes through major phytoplankton groups during the spring bloom and post-bloom in the Northwestern Mediterranean Sea. Deep Sea Res. Part I Oceanogr. Res. Pap. 57, 486–500. 10.1016/j.dsr.2009.12.013

[B48] Hansen H. P. Koroleff F. (2007). Determination of nutrients, in Methods of Seawater Analysis, eds Grasshoff K. Kremling K. Ehrhardt M. (John Wiley & Sons), 159–228. 10.1002/9783527613984.ch10

[B49] Hewson I. Fuhrman J. A. (2007). Characterization of lysogens in bacterioplankton assemblages of the southern California borderland. Microb. Ecol. 53, 631–638. 10.1007/s00248-006-9148-3 17345141

[B50] James A. K. Passow U. Brzezinski M. A. Parsons R. J. Trapani J. N. Carlson C. A. (2017). Elevated pCO_2_ enhances bacterioplankton removal of organic carbon. PLoS ONE 12:e0173145. 10.1371/journal.pone.0173145 28257422 PMC5336268

[B51] Joint I. Doney S. C. Karl D. M. (2011). Will ocean acidification affect marine microbes[quest]. ISME J. 5, 1–7. 10.1038/ismej.2010.79 20535222 PMC3105673

[B52] Joos F. Spahni R. (2008). Rates of change in natural and anthropogenic radiative forcing over the past 20,000 years. Proc. Natl. Acad. Sci. U.S.A. 105, 1425 LP−1430. 10.1073/pnas.0707386105 18252830 PMC2234160

[B53] Kim J.-M. Lee K. Shin K. Yang E. J. Engel A. Karl D. M.. (2011). Shifts in biogenic carbon flow from particulate to dissolved forms under high carbon dioxide and warm ocean conditions. Geophys. Res. Lett. 38:L08612. 10.1029/2011GL047346

[B54] Kirchman D. K'Nees E. Hodson R. (1985). Leucine incorporation and its potential as a measure of protein synthesis by bacteria in natural aquatic systems. Appl. Env. Microbiol. 49, 599–607. 10.1128/AEM.49.3.599-607.1985 3994368 PMC373556

[B55] Knowles B. Silveira C. B. Bailey B. A. Barott K. Cantu V. A. Cobián-Güemes A. G.. (2016). Lytic to temperate switching of viral communities. Nature 531, 466–470. 10.1038/nature17193 26982729

[B56] Krause E. Wichels A. Giménez L. Lunau M. Schilhabel M. B. Gerdts G. (2012). Small changes in pH have direct effects on marine bacterial community composition: a microcosm approach. PLoS ONE 7:e47035. 10.1371/journal.pone.0047035 23071704 PMC3469576

[B57] Larsen A. Castberg T. Sandaa R. A. Brussaard C. P. D. Egge J. K. Heldal M.. (2001). Population dynamics and diversity of phytoplankton, bacteria and viruses in a seawater enclosure. Mar. Ecol. Prog. Ser. 221, 47–57. 10.3354/meps221047

[B58] Larsen A. Gro A. Flaten F. Sandaa R. A. Castberg T. Thyrhaug R.. (2004). Spring phytoplankton bloom dynamics in Norwegian coastal waters: microbial community succession and diversity. Limnol. Oceanogr. 49, 180–190. 10.4319/lo.2004.49.1.0180

[B59] Larsen J. B. Larsen A. Thyrhaug R. Bratbak G. Sandaa R. A. (2008). Response of marine viral populations to a nutrient induced phytoplankton bloom at different pCO_2_ levels. Biogeosciences 5, 523–533. 10.5194/bg-5-523-2008

[B60] Lee S. Fuhrman J. E. (1987). Relationship between biovolume and biomass of naturally derived marine bacterioplankton. Appl. Environ. Microbiol. 53, 1298–1303. 10.1128/AEM.53.6.1298-1303.1987 16347362 PMC203858

[B61] Lindh M. V. Riemann L. Baltar F. Romero-Oliva C. Salomon P. S. Granéli E.. (2013). Consequences of increased temperature and acidification on bacterioplankton community composition during a mesocosm spring bloom in the Baltic Sea. Environ. Microbiol. Rep. 5, 252–262. 10.1111/1758-2229.12009 23584969

[B62] Maat D. S. Crawfurd K. J. Timmermans K. R. Brussaard C. P. D. (2014). Elevated CO_2_ and phosphate limitation favor *Micromonas pusilla* through stimulated growth and reduced viral impact. Appl. Environ. Microbiol. 80, 3119 LP−3127. 10.1128/AEM.03639-13 24610859 PMC4018922

[B63] Malits A. Christaki U. Obernosterer I. Weinbauer M. G. G. (2014). Enhanced viral production and virus-mediated mortality of bacterioplankton in a natural iron-fertilized bloom event above the Kerguelen Plateau. Biogeosciences 11, 6841–6853. 10.5194/bg-11-6841-2014

[B64] Malits A. Weinbauer M. G. G. (2009). Effect of turbulence and viruses on prokaryotic cell size, production and diversity. Aquat. Microb. Ecol. 54, 243–254. 10.3354/ame01274

[B65] Malone T. C. Newton A. (2020). The globalization of cultural eutrophication in the coastal ocean: causes and consequences. Front. Mar. Sci. 7:670. 10.3389/fmars.2020.00670

[B66] Mari X. Kerros M. E. Weinbauer M. G. (2007). Virus attachment to transparent exopolymeric particles along trophic gradients in the southwestern lagoon of New Caledonia. Appl. Env. Microbiol. 73, 5245–5252. 10.1128/AEM.00762-07 17586679 PMC1950989

[B67] Marie D. Brussaard C. P. D. Thyrhaug R. Bratbak G. Vaulot D. (1999). Enumeration of marine viruses in culture and natural samples by flow cytometry. Appl. Env. Microbiol. 65, 45–52. 10.1128/AEM.65.1.45-52.1999 9872758 PMC90981

[B68] Marie D. Partensky F. Jacquet S. Vaulot D. (1997). Enumeration and cell cycle analysis of natural populations of marine picoplankton by flow cytometry using the nucleic acid stain SYBR green I. Appl. Env. Microbiol. 63, 186–193. 10.1128/AEM.63.1.186-193.1997 16535483 PMC1389098

[B69] Marie D. Partensky F. Vaulot D. Brussaard C. (2001). Enumeration of phytoplankton, bacteria, and viruses in marine samples. Curr. Protoc. Cytom. Chapter 11:Unit 11.11. 10.1002/0471142956.cy1111s10 18770685

[B70] Martinez J. M. Swan B. K. Wilson W. H. (2014). Marine viruses, a genetic reservoir revealed by targeted viromics. ISME J. 8, 1079–1088. 10.1038/ismej.2013.214 24304671 PMC3996692

[B71] Meakin N. G. Wyman M. (2011). Rapid shifts in picoeukaryote community structure in response to ocean acidification. ISME J. 5, 1397–1405. 10.1038/ismej.2011.18 21412344 PMC3160676

[B72] Middelboe M. (2000). Bacterial growth rate and marine virus-host dynamics. Microb. Ecol. 40, 114–124. 10.1007/s002480000050 11029080

[B73] Middelboe M. Lyck P. G. (2002). Regeneration of dissolved organic matter by viral lysis in marine microbial communities. Aquat. Microb. Ecol. 27, 187–194. 10.3354/ame027187

[B74] Motegi C. Tanaka T. Piontek J. Brussaard C. P. D. Gattuso J. P. Weinbauer M. G. (2013). Effect of CO_2_ enrichment on bacterial metabolism in an Arctic fjord. Biogeosciences 10, 3285–3296. 10.5194/bg-10-3285-2013

[B75] Murray A. G. Eldridge P. M. (1994). Marine viral ecology: incorporation of bacteriophage into the microbial planktonic food web paradigm. J. Plankton Res. 16, 627–641. 10.1093/plankt/16.6.627

[B76] Newbold L. K. Oliver A. E. Booth T. Tiwari B. Desantis T. Maguire M.. (2012). The response of marine picoplankton to ocean acidification. Env. Microbiol. 14, 2293–2307. 10.1111/j.1462-2920.2012.02762.x 22591022

[B77] Oliver A. E. Newbold L. K. Whiteley A. S. van der Gast C. J. (2014). Marine bacterial communities are resistant to elevated carbon dioxide levels. Environ. Microbiol. Rep. 6, 574–582. 10.1111/1758-2229.12159 25756110

[B78] Ortega-Retuerta E. Marrasé C. Muñoz-Fernández A. Sala M. M. Simó R. Gasol J. M. (2018). Seasonal dynamics of transparent exopolymer particles (TEP) and their drivers in the coastal NW Mediterranean Sea. Sci. Total Environ. 631–632, 180–190. 10.1016/j.scitotenv.2018.02.341 29525702

[B79] Paul A. J. Bach L. T. Schulz K. G. Boxhammer T. Czerny J. Achterberg E. P.. (2015). Effect of elevated CO_2_ on organic matter pools and fluxes in a summer Baltic Sea plankton community. Biogeosciences. 12, 6181–6203. 10.5194/bg-12-6181-2015

[B80] Paul J. H. Weinbauer M. G. (2010). Detection of lysogeny in marine environments, in Manual of Aquatic Viral Ecology, eds Suttle C. Wilhelm S. W. Weinbauer M. G. (Waco: ASLO), 1–8. 10.4319/mave.2010.978-0-9845591-0-7.30

[B81] Pérez F. F. Ríos A. F. Rellán T. Álvarez M. (2000). Improvements in a fast potentiometric seawater alkalinity determination. Ciencias Mar. 26, 463–478. 10.7773/cm.v26i3.592

[B82] Piontek J. Borchard C. Sperling M. Schulz K. G. Riebesell U. Engel A. (2013). Response of bacterioplankton activity in an Arctic fjord system to elevated pCO_2_: results from a mesocosm perturbation study. Biogeosciences 10, 297–314. 10.5194/bg-10-297-2013

[B83] Poorvin L. Rinta-Kanto J. M. Hutchins D. A. Wilhelm S. W. (2004). Viral release of iron and its bioavailability to marine plankton. Limnol. Oceanogr. 49, 1734–1741. 10.4319/lo.2004.49.5.1734

[B84] Provoost P. van Heuven S. Soetaert K. Laane R. W. P. M. Middelburg J. J. (2010). Seasonal and long-term changes in pH in the Dutch coastal zone. Biogeosciences 7, 3869–3878. 10.5194/bg-7-3869-2010

[B85] Rabalais N. N. Turner R. E. Díaz R. J. Justić D. (2009). Global change and eutrophication of coastal waters. ICES J. Mar. Sci. 66, 1528–1537. 10.1093/icesjms/fsp047

[B86] Raven J. A. Giordano M. Beardall J. Maberly S. C. (2012). Algal evolution in relation to atmospheric CO_2_: carboxylases, carbon-concentrating mechanisms and carbon oxidation cycles. Philos. Trans. R. Soc. Lond. B. Biol. Sci. 367, 493–507. 10.1098/rstb.2011.0212 22232762 PMC3248706

[B87] Riebesell U. Bellerby R. G. J. Grossart H. P. Thingstad F. (2008). Mesocosm CO_2_ perturbation studies: from organism to community level. Biogeosciences 5, 1157–1164. 10.5194/bg-5-1157-2008

[B88] Rochelle-Newall E. Delille B. Frankignoulle M. Gattuso J. P. Jacquet S. Riebesell U.. (2004). Chromophoric dissolved organic matter in experimental mesocosms maintained under different pCO_2_ levels. Mar. Ecol. Prog. Ser. 272, 25–31. 10.3354/meps272025

[B89] Roy A. S. Gibbons S. M. Schunck H. Owens S. Caporaso J. G. Sperling M.. (2013). Ocean acidification shows negligible impacts on high-latitude bacterial community structure in coastal pelagic mesocosms. Biogeosciences 10, 555–566. 10.5194/bg-10-555-2013

[B90] Ruardij P. Veldhuis M. J. W. Brussaard C. P. D. (2005). Modeling the bloom dynamics of the polymorphic phytoplankter *Phaeocystis globosa*: impact of grazers and viruses. Harmful Algae 4, 941–963. 10.1016/j.hal.2004.12.011

[B91] Sabine C. L. Feely R. A. Gruber N. Key R. M. Lee K. Bullister J. L.. (2004). The oceanic sink for anthropogenic CO_2_. Science 305, 367–371. 10.1126/science.1097403 15256665

[B92] Sala M. M. Aparicio F. L. Balagué V. Boras J. A. Borrull E. Cardelús C.. (2016). Contrasting effects of ocean acidification on the microbial food web under different trophic conditions. ICES J. Mar. Sci. 73, 670–679. 10.1093/icesjms/fsv130

[B93] Sandaa R.-A. A. Gomez-Consarnau L. Pinhassi J. Riemann L. Malits A. Weinbauer M. G. G.. (2009). Viral control of bacterial biodiversity - evidence from a nutrient-enriched marine mesocosm experiment. Env. Microbiol. 11, 2585–2597. 10.1111/j.1462-2920.2009.01983.x 19558511

[B94] Schulz K. G. Bach L. T. Bellerby R. G. J. Bermúdez R. Büdenbender J. Boxhammer T.. (2017). Phytoplankton blooms at increasing levels of atmospheric carbon dioxide: experimental evidence for negative effects on prymnesiophytes and positive on small picoeukaryotes. Front. Mar. Sci. 4:64. 10.3389/fmars.2017.00064

[B95] Sheik A. R. Brussaard C. P. D. Lavik G. Lam P. Musat N. Krupke A.. (2014). Responses of the coastal bacterial community to viral infection of the algae *Phaeocystis globosa*. ISME J. 8, 212–225. 10.1038/ismej.2013.135 23949664 PMC3869014

[B96] Sherr B. F. del Giorgio P. A. Sherr E. B. (1999). Estimating abundance and single-cell characteristics of actively respiring bacteria via the redox dye CTC. Aquat. Microb. Ecol. 18, 117–131. 10.3354/ame018117

[B97] Sieracki M. E. Cucci T. L. Nicinski J. (1999). Flow cytometric analysis of 5-cyano-2,3-ditolyl tetrazolium chloride activity of marine bacterioplankton in dilution cultures. Appl. Env. Microbiol. 65, 2409–2417. 10.1128/AEM.65.6.2409-2417.1999 10347021 PMC91356

[B98] Simek K. Pernthaler J. Weinbauer M. G. Hornak K. Dolan J. R. Nedoma J.. (2001). Changes in bacterial community composition and dynamics and viral mortality rates associated with enhanced flagellate grazing in a mesoeutrophic reservoir. Appl. Env. Microbiol. 67, 2723–2733. 10.1128/AEM.67.6.2723-2733.2001 11375187 PMC92931

[B99] Simon M. Azam F. (1989). Protein content and protein synthesis rates of planktonic marine bacteria. Mar. Ecol. Prog. Ser. 51, 201–213.

[B100] Smith D. C. Azam F. (1992). A simple, economical method for measuring bacterial protein synthesis rates in seawater using 3H-leucine. Mar. Microb. Food Webs 6, 107–114.

[B101] Sperling M. Piontek J. Gerdts G. Wichels A. Schunck H. Roy A. S.. (2013). Effect of elevated CO_2_ on the dynamics of particle-attached and free-living bacterioplankton communities in an Arctic fjord. Biogeosciences 10, 181–191. 10.5194/bg-10-181-2013

[B102] Spilling K. Paul A. J. Virkkala N. Hastings T. Lischka S. Stuhr A.. (2016). Ocean acidification decreases plankton respiration: evidence from a mesocosm experiment. Biogeosciences 13, 4707–4719. 10.5194/bg-13-4707-2016

[B103] Steward G. F. Smith D. C. Azam F. (1996). Abundance and production of bacteria and viruses in the Bering and Chukchi Seas. Mar. Ecol. Prog. Ser. 131, 287–300. 10.3354/meps131287

[B104] Suttle C. A. (2000). Ecological, evolutionary, and geochemical consequences of viral infection of cyanobacteria and eukaryotic algae, in Viral Ecology, ed Hurst C. J. (Cambridge: Academic Press), 247–296. 10.1016/B978-012362675-2/50007-0

[B105] Suttle C. A. (2005). Viruses in the sea. Nature 437, 356–361. 10.1038/nature04160 16163346

[B106] Suttle C. A. (2007). Marine viruses–major players in the global ecosystem. Nat. Rev. Microbiol. 5, 801–812. 10.1038/nrmicro1750 17853907

[B107] Traving S. J. Clokie M. R. Middelboe M. (2013). Increased acidification has a profound effect on the interactions between the cyanobacterium *Synechococcus* sp. WH7803 and its viruses. FEMS Microbiol. Ecol. 87, 133–141. 10.1111/1574-6941.12199 24003947

[B108] Tsiola A. Pitta P. Giannakourou A. Bourdin G. Marro S. Maugendre L.. (2017). Ocean acidification and viral replication cycles: frequency of lytically infected and lysogenic cells during a mesocosm experiment in the NW Mediterranean Sea. Estuar. Coast. Shelf Sci. 186, 139–151. 10.1016/j.ecss.2016.05.003

[B109] Unrein F. Gasol J. M. Not F. Forn I. Massana R. (2014). Mixotrophic haptophytes are key bacterial grazers in oligotrophic coastal waters. ISME J. 8, 164–176. 10.1038/ismej.2013.132 23924785 PMC3869011

[B110] Vaqué D. Lara E. Arrieta J. M. Holding J. Sà E. L. Hendriks I. E.. (2019). Warming and CO_2_ enhance arctic heterotrophic microbial activity. Front. Microbiol. 10:494. 10.3389/fmicb.2019.00494 30949141 PMC6436474

[B111] Weinbauer M. G. (2004). Ecology of prokaryotic viruses. FEMS Microbiol. Rev. 28, 127–181. 10.1016/j.femsre.2003.08.001 15109783

[B112] Weinbauer M. G. Arrieta J. M. Griebler C. Herndl G. J. (2009). Enhanced viral production and infection of bacterioplankton during an iron induced phytoplankton bloom in the Southern Ocean. Limnol. Oceanogr. 54, 774–784. 10.4319/lo.2009.54.3.0774

[B113] Weinbauer M. G. Bonilla-Findji O. Chan A. M. Dolan J. R. Short S. M. Simek K.. (2011). *Synechococcus* growth in the ocean may depend on the lysis of heterotrophic bacteria. J. Plankton Res. 33, 1465–1476. 10.1093/plankt/fbr041

[B114] Weinbauer M. G. Brettar I. Höfle M. G. (2003a). Lysogeny and virus-induced mortality of bacterioplankton in surface, deep, and anoxic marine waters. Limnol. Oceanogr. 48, 1457–1465. 10.4319/lo.2003.48.4.1457

[B115] Weinbauer M. G. Christaki U. Nedoma J. Simek K. (2003b). Comparing the effects of resource enrichment and grazing on viral production in a meso-eutrophic reservoir. Aquat. Microb. Ecol. 31, 137–144. 10.3354/ame031137

[B116] Weinbauer M. G. Rassoulzadegan F. (2004). Are viruses driving microbial diversification and diversity? Environ. Microbiol. 6, 1–11. 10.1046/j.1462-2920.2003.00539.x 14686936

[B117] Weinbauer M. G. Rowe J. M. Wilhelm S. W. (2010). Determining rates of virus production in aquatic systems by the virus reduction approach, in Manual of Aquatic Viral Ecology, eds Suttle C. Wilhelm S. W. Weinbauer M. G. (Waco: ASLO), 1–8. 10.4319/mave.2010.978-0-9845591-0-7.1

[B118] Wells L. E. Deming J. W. (2006). Significance of bacterivory and viral lysis in bottom waters of Franklin Bay, Canadian Arctic, during winter. Aquat. Microb. Ecol. 43, 209–221. 10.3354/ame043209

[B119] Wilhelm S. W. Suttle C. A. (1999). Virus and nutrient cycles in the sea. Bioscience 49, 781–787. 10.2307/1313569

[B120] Wilson W. H. Mann N. H. (1997). Lysogenic and lytic viral production in marine microbial communities. Aquat. Microb. Ecol. 13, 95–100. 10.3354/ame013095

[B121] Worden A. Z. Follows M. J. Giovannoni S. J. Wilken S. Zimmerman A. E. Keeling P. J. (2015). Rethinking the marine carbon cycle: factoring in the multifarious lifestyles of microbes. Science 347:1257594. 10.1126/science.1257594 25678667

[B122] Yentsch C. S. Menzel D. W. (1963). A method for the determination of phytoplankton chlorophyll and phaeophytin by fluorescence. Deep Sea Res. 10, 221–231. 10.1016/0011-7471(63)90358-9

[B123] Yoshimura T. Suzuki K. Kiyosawa H. Ono T. Hattori H. Kuma K.. (2013). Impacts of elevated CO_2_ on particulate and dissolved organic matter production: microcosm experiments using iron-deficient plankton communities in open subarctic waters. J. Oceanogr. 69, 601–618. 10.1007/s10872-013-0196-2

[B124] Zeebe R. E. Ridgwell A. Zachos J. C. (2016). Anthropogenic carbon release rate unprecedented during the past 66 million years. Nat. Geosci. 9:325. 10.1038/ngeo2681

[B125] Zhang R. Xia X. Lau S. C. K. Motegi C. Weinbauer M. G. Jiao N. (2013). Response of bacterioplankton community structure to an artificial gradient of pCO_2_ in the Arctic Ocean. Biogeosciences 10, 3679–3689. 10.5194/bg-10-3679-2013

