# Supplementary material for: Viral-Mediated Microbe Mortality Modulated by Ocean Acidification and Eutrophication: Consequences for the Carbon Fluxes Through the Microbial Food Web
Source: Front Microbiol. 2021 Apr 14;12:635821. doi: 10.3389/fmicb.2021.635821 (PMC8079731; doi:10.3389/fmicb.2021.635821)
Supplement: Supplementary file 1 [file Data_Sheet_1.docx]

Figure S1. Flow cytometric plot of side scatter (SSC) versus SYBR green fluorescence signal (FL1-H) from a 10 times diluted sample of the NB treatment of experiment WINTER. Four different populations of virus-like particles (VLP) can be distinguished: low fluorescence VLP (LOW), medium fluorescence VLP (MEDIUM), high fluorescence VLP (HIGH) and very high fluorescence VLP (VERY HIGH). Bacterial populations (LNA and HNA, see text) can be discriminated.

Figure S2. Temporal dynamics of *Prochlorococcus* *sp*. during the experiments WINTER (A) and SUMMER (B). Bars indicate the maximum and minimum values of experimental duplicates; when not visible they are contained within the symbol. KB: control, KA: lowered pH, NB: nutrient amended, NA: nutrient amended and lowered pH.

TABLE S1: Experiment WINTER. Nonparametric Spearman rank correlation matrix for chlorophyll a, picophytoplankton, bacterial and viral parameters. Probabilities in the upper triangle and Spearman's correlation coefficient ρ in the lower triangle. K, control; N nutrient amendment, VLP, total abundance of virus-like particles (VLP); LOW, MED, HIGH, VERY: VLP subpopulations with low, medium, high or very high fluorescence in the cytometric signature, respectively; VP_L_, lytic viral production; VMM, viral mediated loss of bacterial standing stock per day: BA, bacterial abundance; LNA and HNA, bacteria with low and high fluorescence in the flow cytometric signature, respectively; BP, bacterial heterotrophic production; CTC+, the percentage of CTC labelled cells; chl *a*, chlorophyll *a*; Syn, Synechococcus; Pro, Prochlorococcus; S pico, small autotrophic picoeukaryotes; L pico, large autotrophic picoeukaryotes.

| K | VLP | LOW | MED | HIGH | VERY | VP_L_ | VMM | BA | LNA | HNA | BP | CTC+ | chl *a* | Syn | Pro | S pico | L pico |
| --- | --- | --- | --- | --- | --- | --- | --- | --- | --- | --- | --- | --- | --- | --- | --- | --- | --- |
| VLP | 1 | *** | ns | ns | ns | 0.004 | ns | 0.004 | 0.018 | 0.006 | 0.010 | ns | 0.043 | ns | ns | 0.033 | ns |
| LOW | **0.792** | 1 | 0.014 | ns | *** | 0.045 | *** | ns | 0.014 | ns | ns | ns | 0.0003 | 0.003 | 0.034 | ns | ns |
| MED | 0.146 | **-0.407** | 1 | *** | *** | ns | 0.011 | *** | ns | *** | *** | 0.0002 | 0.004 | *** | *** | *** | *** |
| HIGH | 0.182 | -0.291 | **0.806** | 1 | *** | ns | *** | *** | ns | *** | 0.0003 | *** | ns | *** | *** | *** | *** |
| VERY | 0.267 | **0.703** | **-0.850** | **-0.654** | 1 |  |  | 0.003 | ns | 0.001 | 0.004 | 0.019 | *** | *** | *** | *** | *** |
| VP_L_ | **0.700** | **0.524** | 0.415 | 0.044 |  | 1 | ns | *** | *** | *** | *** | ns | ns | ns | *** | ns | ns |
| VMM | 0.295 | **0.840** | **-0.633** | **-0.873** |  | 0.332 | 1 | ns | ns | ns | ns | ns | ns | 0.0035 | ns | 0.011 | 0.003 |
| BA | **0.470** | 0.079 | **0.672** | **0.608** | **-0.502** | **0.829** | -0.164 | 1 | 0.020 | *** | *** | 0.0002 | ns | 0.003 | *** | *** | *** |
| LNA | **0.393** | **0.407** | -0.156 | 0.089 | 0.336 | **0.938** | 0.207 | **0.387** | 1 | 0.032 | ns | ns | 0.0002 | ns | ns | ns | *** |
| HNA | **0.451** | 0.044 | **0.702** | **0.626** | **-0.542** | **0.797** | -0.164 | **0.994** | **0.358** | 1 | *** | 0.0002 | ns | 0.001 | *** | *** | *** |
| BP | **0.425** | -0.010 | **0.743** | **0.564** | **-0.492** | **0.829** | -0.164 | **0.781** | 0.093 | **0.784** | 1 | *** | ns | *** | *** | *** | *** |
| CTC+ | 0.052 | -0.304 | **0.690** | **0.720** | **-0.474** | 0.458 | -0.404 | **0.684** | -0.120 | **0.688** | **0.717** | 1 | ns | 0.020 | 0.0002 | ns | *** |
| chl *a* | **0.340** | **0.574** | **-0.469** | -0.159 | **0.634** | 0.044 | 0.229 | -0.013 | **0.583** | -0.057 | -0.214 | -0.268 | 1 | *** | 0.010 | ns | ns |
| Syn | 0.005 | **-0.478** | **0.817** | **0.667** | **-0.776** | 0.218 | **-0.546** | **0.488** | -0.077 | **0.512** | **0.614** | **0.472** | **-0.616** | 1 | *** | *** | *** |
| Pro | 0.188 | **-0.354** | **0.925** | **0.816** | **-0.811** | **0.949** | 0.264 | **0.692** | 0.026 | **0.712** | **0.727** | **0.681** | **-0.424** | **0.909** | 1 | *** | *** |
| S pico | **0.356** | -0.151 | **0.872** | **0.826** | **-0.758** | 0.415 | **-0.633** | **0.753** | -0.005 | **0.769** | **0.706** | **0.817** | -0.328 | **0.738** | **0.902** | 1 | *** |
| L pico | 0.289 | -0.205 | **0.884** | **0.794** | **-0.700** | 0.281 | **-0.777** | **0.773** | -0.036 | **0.789** | **0.766** | **0.764** | -0.195 | **0.624** | **0.830** | **0.849** | 1 |
| N | VLP | LOW | MED | HIGH | VERY | VP_L_ | VMM | BA | LNA | HNA | BP | CTC+ | chl *a* | Syn | Pro | S pico | L pico |
| VLP | 1 | *** | *** | *** | *** | ns | ns | ns | *** | ns | ns | ns | *** | ns | ns | ns | 0.0003 |
| LOW | **0.973** | 1 | 0.0006 | *** | *** | ns | ns | ns | *** | ns | ns | ns | *** | ns | 0.020 | 0.040 | 0.001 |
| MED | **0.641** | **0.545** | 1 | 0.027 | ns | ns | 0.037 | ns | 0.008 | ns | 0.033 | ns | ns | ns | ns | ns | ns |
| HIGH | **0.709** | **0.619** | **0.340** | 1 | ns | ns | 0.033 | 0.003 | *** | 0.007 | ns | ns | *** | ns | ns | ns | *** |
| VERY | **0.776** | **0.814** | 0.322 | 0.341 | 1 |  |  | ns | *** | ns | ns | ns | *** | ns | *** | *** | ns |
| VP_L_ | -0.501 | -0.164 | -0.558 | -0.436 |  | 1 | ns | ns | ns | ns | 0.017 | ns | ns | ns | 0.021 | ns | ns |
| VMM | 0.295 | 0.323 | **-0.633** | **-0.642** |  | 0.455 | 1 | 0.049 | ns | 0.049 | ns | ns | 0.049 | ns | ns | ns | 0.037 |
| BA | 0.236 | 0.149 | 0.244 | **0.475** | -0.181 | 0.248 | **-0.605** | 1 | ns | *** | 0.006 | 0.020 | ns | 0.0007 | *** | *** | 0.010 |
| LNA | **0.769** | **0.735** | **0.435** | **0.648** | **0.714** | -0.400 | -0.183 | 0.309 | 1 | ns | ns | ns | *** | ns | ns | ns | *** |
| HNA | 0.186 | 0.102 | 0.200 | **0.439** | -0.233 | 0.248 | **-0.605** | **0.994** | 0.254 | 1 | 0.005 | 0.011 | ns | 0.0004 | *** | *** | 0.015 |
| BP | -0.022 | -0.089 | **0.357** | -0.115 | -0.134 | **-0.698** | -0.183 | **0.453** | 0.038 | **0.457** | 1 | ns | ns | ns | ns | ns | ns |
| CTC+ | -0.107 | -0.176 | -0.101 | 0.304 | -0.304 | 0.164 | -0.323 | **0.470** | -0.110 | **0.509** | 0.391 | 1 | ns | 0.003 | 0.001 | 0.0003 | ns |
| chl *a* | **0.753** | **0.804** | 0.177 | **0.607** | **0.790** | -0.248 | **0.605** | -0.051 | **0.623** | -0.079 | -0.246 | 0.176 | 1 | ns | 0.018 | ns | *** |
| Syn | 0.120 | 0.102 | -0.062 | 0.176 | -0.133 | 0.501 | -0.295 | **0.541** | 0.023 | **0.560** | 0.228 | **0.582** | 0.086 | 1 | *** | *** | ns |
| Pro | -0.287 | **-0.386** | -0.132 | 0.208 | **-0.683** | **0.878** | 0.098 | **0.690** | -0.174 | **0.713** | 0.247 | **0.624** | **-0.393** | **0.620** | 1 | *** | ns |
| S pico | -0.272 | **-0.343** | -0.278 | 0.265 | **-0.642** | 0.501 | -0.295 | **0.633** | -0.218 | **0.669** | 0.150 | **0.681** | -0.194 | **0.604** | **0.818** | 1 | 0.029 |
| L pico | **0.571** | **0.523** | 0.485 | **0.855** | 0.332 | -0.089 | **-0.634** | **0.421** | **0.620** | **0.403** | -0.231 | 0.299 | **0.701** | 0.270 | 0.178 | **0.365** | 1 |

*** *P* = < 0.0001

TABLE S2: Experiment SUMMER. Nonparametric Spearman rank correlation matrix for chlorophyll a, picophytoplankton, bacterial and viral parameters. Probabilities in the upper triangle and Spearman's correlation coefficient ρ in the lower triangle. For abbreviations see TABLE S1.

| K | VLP | LOW | MED | HIGH | VP_L_ | VMM | BA | LNA | HNA | BP | CTC+ | chl *a* | Syn | Pro | S pico | L pico |
| --- | --- | --- | --- | --- | --- | --- | --- | --- | --- | --- | --- | --- | --- | --- | --- | --- |
| VLP | 1 | *** | *** | *** | ns | 0.009 | *** | 0.034 | 0.027 | 0.001 | 0.011 | 0.001 | ns | ns | *** | ns |
| LOW | **0.932** | 1 | *** | *** | ns | 0.009 | *** | ns | *** | 0.007 | 0.049 | 0.015 | ns | ns | *** | ns |
| MED | **0.861** | **-0.407** | 1 | *** | ns | 0.017 | 0.010 | 0.004 | ns | 0.0005 | 0.036 | 0.004 | ns | ns | *** | ns |
| HIGH | **0.858** | **0.716** | **0.867** | 1 | ns | 0.009 | 0.0005 | *** | ns | 0.0003 | 0.021 | 0.001 | ns | ns | *** | 0.019 |
| VP_L_ | -0.086 | -0.086 | -0.173 | -0.086 | 1 | 0.017 | ns | 0.021 | ns | ns | 0.017 | ns | ns | ns | ns | ns |
| VMM | **-0.713** | **-0.713** | **-0.669** | **-0.713** | **0.671** | 1 | 0.009 | ns | ns | ns | 0.003 | 0.009 | ns | ns | 0.017 | ns |
| BA | **0.701** | **0.836** | **0.423** | **0.550** | -0.086 | **-0.713** | 1 | ns | *** | ns | ns | ns | 0.004 | ns | 0.0007 | ns |
| LNA | **-0.418** | -0.214 | **-0.540** | **-0.756** | **0.878** | 0.683 | -0.065 | 1 | ns | *** | ns | ns | 0.004 | 0.006 | *** | ns |
| HNA | **0.434** | **0.700** | 0.255 | 0.110 | 0.527 | -0.316 | **0.853** | 0.211 | 1 | ns | ns | ns | ns | ns | ns | ns |
| BP | **-0.513** | **-0.441** | **-0.548** | **-0.571** | -0.126 | -0.291 | -0.152 | **0.748** | -0.069 | 1 | ns | ns | *** | *** | *** | ns |
| CTC+ | **-0.464** | **-0.369** | **-0.391** | **-0.427** | **0.669** | **0.777** | -0.182 | 0.216 | 0.212 | 0.313 | 1 | 0.037 | ns | ns | ns | 0.019 |
| chl *a* | **0.514** | **0.408** | **0.477** | **0.522** | -0.086 | **-0.713** | 0.223 | -0.369 | 0.135 | -0.236 | **-0.395** | 1 | ns | ns | ns | 0.0002 |
| Syn | 0.031 | 0.199 | -0.239 | -0.118 | -0.216 | -0.259 | **0.473** | **0.547** | 0.189 | **0.615** | 0.016 | -0.140 | 1 | 0.001 | ns | ns |
| Pro | -0.156 | -0.168 | -0.123 | -0.266 | 0.022 | -0.389 | 0.006 | **0.531** | 0.087 | **0.722** | 0.190 | -0.060 | **0.527** | 1 | 0.0006 | ns |
| S pico | **-0.777** | **-0.757** | **-0.673** | **0.777** | 0.173 | **0.669** | **-0.537** | **0.755** | -0.216 | **0.774** | 0.256 | -0.317 | 0.238 | **0.553** | 1 | ns |
| L pico | 0.196 | 0.070 | 0.186 | **0.389** | 0.389 | 0.173 | 0.051 | -0.219 | -0.288 | -0.054 | **-0.434** | **0.594** | -0.018 | 0.034 | -0.105 | 1 |
| N | VLP | LOW | MED | HIGH | VP_L_ | VMM | BA | LNA | HNA | BP | CTC+ | chl *a* | Syn | Pro | S pico | L pico |
| VLP | 1 | *** | *** | *** | ns | ns | *** | ns | ns | ns | 0.011 | 0.015 | *** | *** | 0.0002 | 0.002 |
| LOW | **0.924** | 1 | *** | *** | ns | ns | *** | ns | 0.001 | ns | 0.049 | 0.015 | *** | *** | *** | 0.002 |
| MED | **0.926** | **0.825** | 1 | *** | ns | ns | *** | ns | ns | ns | 0.036 | ns | *** | *** | *** | 0.026 |
| HIGH | **0.838** | **0.774** | **0.866** | 1 | ns | ns | *** | 0.003 | ns | ns | 0.021 | 0.001 | *** | 0.0001 | 0.009 | 0.001 |
| VP_L_ | -0.577 | -0.577 | -0.577 | -0.577 | 1 | 0.037 | ns | ns | ns | ns | ns | ns | ns | ns | ns | ns |
| VMM | -0.866 | -0.866 | -0.866 | -0.866 | **0.900** | 1 | ns | ns | ns | ns | ns | ns | ns | ns | ns | ns |
| BA | **0.760** | **0.866** | **0.713** | **0.739** | -0.577 | -0.866 | 1 | ns | *** | ns | ns | 0.017 | *** | *** | *** | 0.005 |
| LNA | -0.309 | 0.034 | -0.312 | **-0.552** | 0.577 | 0.866 | 0.121 | 1 | 0.024 | ns | ns | 0.017 | ns | 0.048 | ns | 0.008 |
| HNA | 0.352 | **0.593** | 0.310 | 0.243 | -0.577 | -0.866 | **0.779** | **0.443** | 1 | 0.011 | ns | ns | *** | *** | 0.001 | ns |
| BP | -0.226 | -0.127 | -0.251 | -0.227 | -0.577 | -0.866 | 0.036 | 0.332 | **0.490** | 1 | ns | ns | ns | ns | ns | ns |
| CTC+ | **-0.433** | **-0.369** | **-0.365** | **-0.443** | 0.577 | 0.866 | -0.182 | 0.216 | 0.212 | 0.313 | 1 | 0.002 | ns | ns | ns | 0.002 |
| chl *a* | **0.403** | **0.402** | 0.252 | **0.512** | 0.577 | 0.866 | **0.396** | **-0.585** | 0.091 | -0.005 | **-0.500** | 1 | ns | ns | ns | *** |
| Syn | **0.807** | **0.878** | **0.766** | **0.718** | -0.577 | -0.866 | **0.911** | 0.064 | **0.762** | -0.036 | -0.197 | 0.270 | 1 | *** | *** | 0.012 |
| Pro | **0.764** | **0.857** | **0.755** | **0.608** | -0.577 | -0.866 | **0.830** | **0.400** | **0.716** | -0.017 | -0.155 | 0.063 | **0.855** | 1 | *** | ns |
| S pico | **-0.583** | **-0.621** | **-0.659** | **-0.436** | 0.577 | 0.866 | **-0.689** | -0.351 | **-0.605** | 0.043 | 0.058 | 0.210 | **-0.756** | **-0.768** | 1 | ns |
| L pico | **0.515** | **0.514** | **0.377** | **0.534** | 0.577 | 0.866 | **0.465** | **-0.522** | 0.119 | 0.020 | **-0.503** | **0.924** | **0.422** | 0.238 | 0.042 | 1 |

*** *P* = < 0.0001
