# Supplementary figures and images for: Viral-Mediated Microbe Mortality Modulated by Ocean Acidification and Eutrophication: Consequences for the Carbon Fluxes Through the Microbial Food Web
Source: Front Microbiol. 2021 Apr 14;12:635821. doi: 10.3389/fmicb.2021.635821 (PMC8079731; doi:10.3389/fmicb.2021.635821)

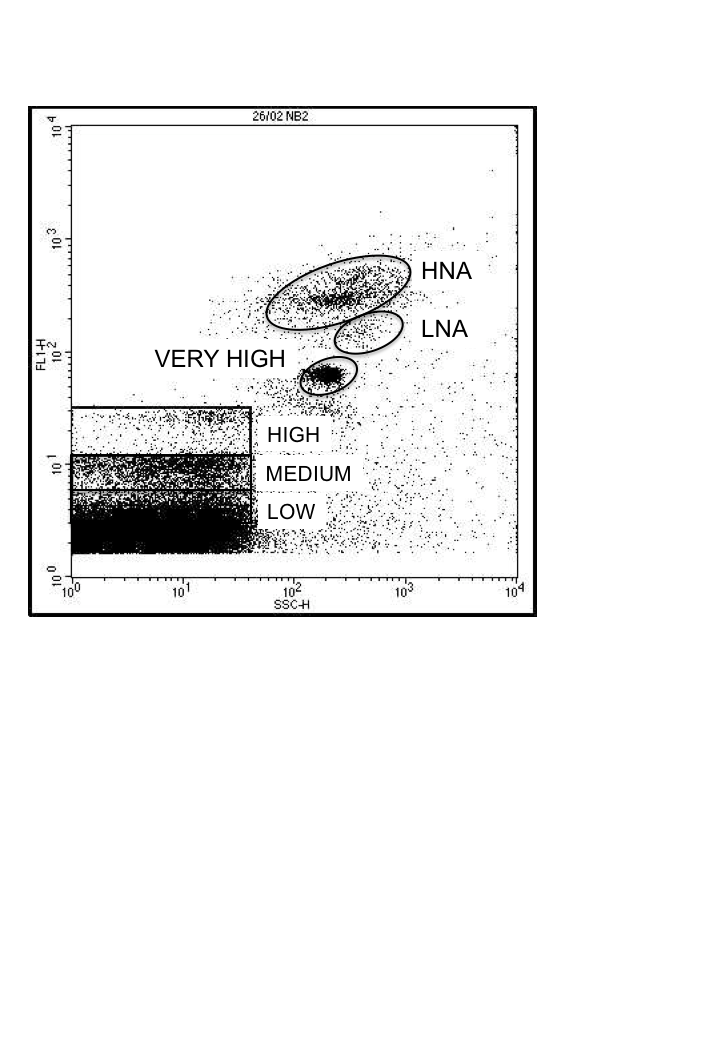

Supplement: Supplementary file 2 [file Image_1.TIFF]

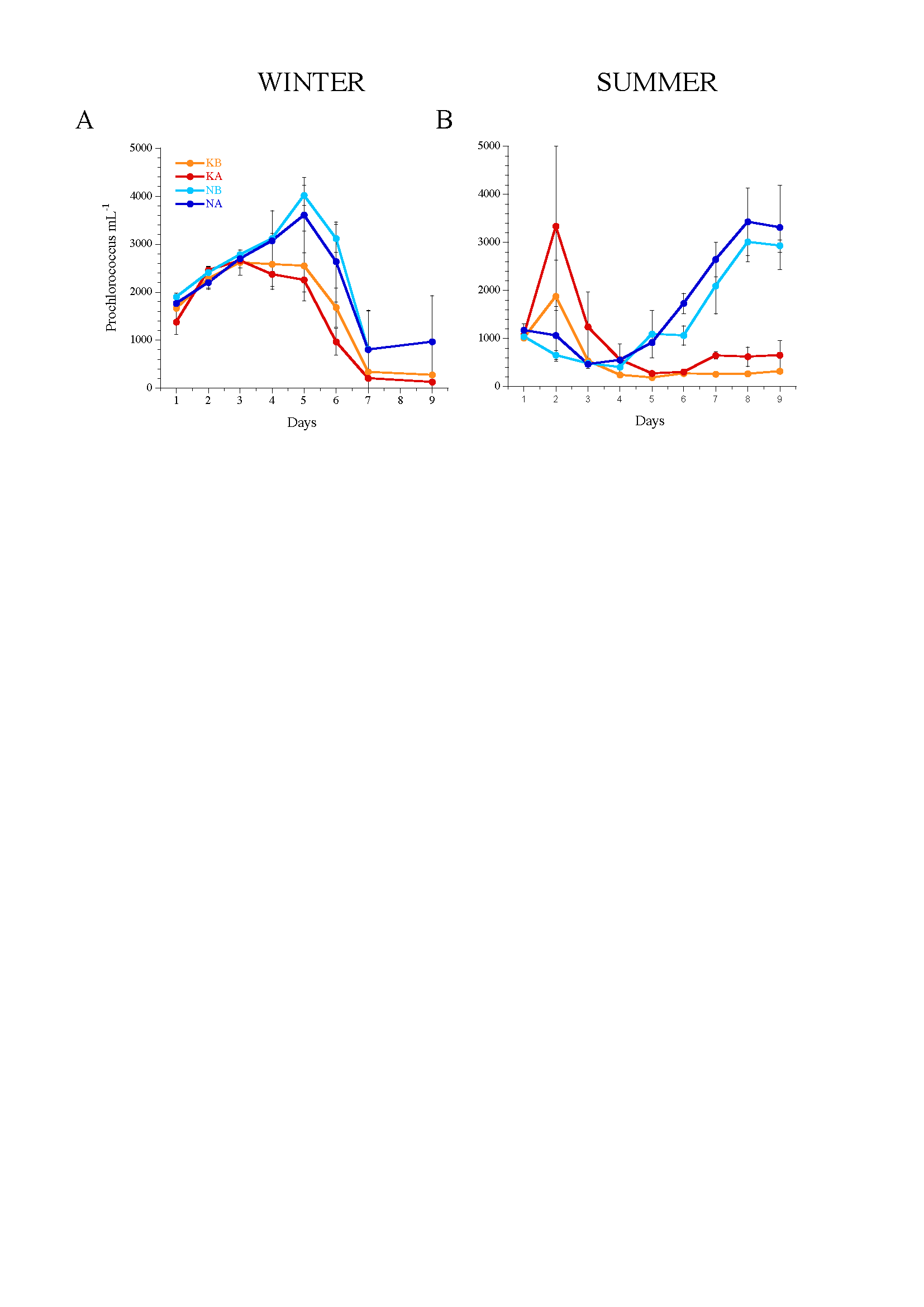

Supplement: Supplementary file 3 [file Image_2.TIFF]
